# Structural and Electronic Reconstruction of Extended Defects in Pnictogen Chalcohalides

**DOI:** 10.1021/acs.jpclett.5c03107

**Published:** 2026-03-04

**Authors:** Thomas Lynch, Cibrán López, Claudio Cazorla, Keith P. McKenna

**Affiliations:** † School of Physics, Engineering and Technology, 8748 University of York, York YO10 5DD, U.K.; ‡ Departament de Física, 16767 Universitat Politècnica de Catalunya, Campus Nord, Jordi Girona 1–3, 08005 Barcelona, Spain; ¶ Research Center in Multiscale Science and Engineering, Universitat Politècnica de Catalunya, Campus Diagonal-Besòs, Av. Eduard Maristany 10-14, 08019 Barcelona, Spain; § Institució Catalana de Recerca i Estudis Avançats (ICREA), Passeig Lluís Companys 23, 08010 Barcelona, Spain

## Abstract

With growing global demand for renewable energy, thin-film photovoltaic technologies are emerging as a promising route to low-cost, scalable solar power. However, for many candidate materials extended defects in polycrystalline thin films are associated with deep gap states that limit carrier lifetimes and reduce device efficiency. Pnictogen chalcohalide semiconductors with the general formula MChX (M = pnictogen, Ch = chalcogen, X = halogen) have been proposed as defect-tolerant alternatives. Using density functional theory, we predict the structure and electronic properties of surface defects for eight pnictogen chalcohalide compounds and analyze their behavior upon surface reconstruction. Our results reveal that, despite the cleavage of covalent bonds, these materials undergo reconstructions that eliminate detrimental gap states. The facile formation of new interchain bonds at the surface preserves the electronic performance of the materials and suggests intrinsic resilience to extended defects. These findings position pnictogen chalcohalides as promising candidates for defect-tolerant, stable, thin-film photovoltaic absorbers.

## Graphical abstract

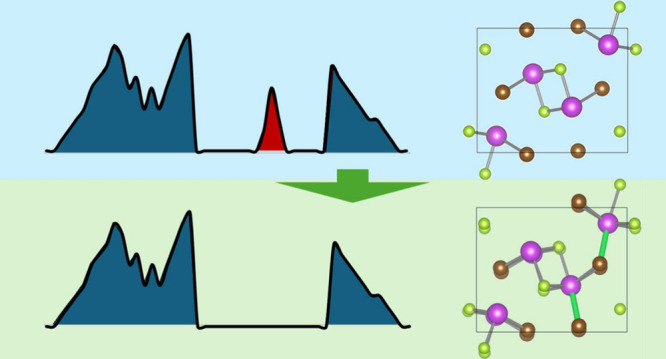

Photovoltaics (PV) are one of the key technologies that can enable our migration toward the generation of clean renewable energy. Monocrystalline silicon dominates the current PV market with a very competitive cost-per-watt compared to alternative energy generation technologies.^1,2^ But there is also considerable research effort directed toward the discovery and optimization of new polycrystalline thin-film solar absorber materials that can be deposited on flexible or lightweight substrates, enabling low-cost, large-area, and versatile solar energy applications as well as combined with complementary absorbers in tandem cells to increase efficiency.^3,4^ In this respect, chalcogenide materials have emerged as a very rich materials design space for solar absorbers, with CdTe the market-leading commercial thin-film PV material and several other compounds (CuZnSnS/Se, CuInGaS/Se and Sb_2_S/Se_3_) with demonstrated efficiencies in excess of 10%.^5–9^ However, a universal challenge is that point defects as well as dangling bonds at extended defects often introduce electronic states deep within the band gap, enhancing nonradiative recombination and limiting efficiency.^10^ For example, in the case of CdTe postgrowth grain boundary treatment with chlorine is required to deliver acceptable performance,^11–13^ but this approach is not straightforward to transfer to other materials. On the other hand, recent first-principles calculations^14,15^ supported by experimental transmission electron microscopy observations^16^ have shown that extended defects in Sb_2_Se_3_ exhibit a remarkable ability to reconstruct, eliminating dangling bonds and the associated deep gap states. Provided point defects and heterointerfaces can be optimized sufficiently for Sb_2_Se_3_ this will permit high-performance polycrystalline thin films that do not need grain boundary treatment, which would be a significant advantage. So far only Sb_2_Se_3_ and the isostructural Sb_2_S_3_ have been predicted to exhibit this behavior. But are there other promising absorber materials that are also tolerant to extended defects and if so what are their common characteristics? Answering this question would be extremely helpful to guide materials discovery as well as steer future efforts on materials and device optimization.

In this letter, we present a first-principles investigation of extended defects in pnictogen chalcohalide materials in order to understand their structure, stability and electronic properties. In particular, we consider materials with the general formula MChX (where M = Bi/Sb, Ch = S/Se and X = I/Br) which have gained attention as potentially defect-tolerant semiconductors for PV applications.^17–25^ These materials are structurally similar to Sb_2_Se_3_ (space group *Pnma*), comprised of one-dimensional (1D) covalently bonded ribbons with open spaces between and also containing cations with lone-pair electrons. Since pnictogen chalcohalide thin films are typically polycrystalline understanding the properties of grain boundary defects is important since they are often performance limiting for PV applications (as in the case of CdTe discussed above). In this letter we use the term “extended defect” in the broadest possible sense to include any disruption of the order of an infinite periodic crystal with extension in one or more dimensions.^26^ Viewed in this way surfaces and grain boundaries are both examples of two-dimensional extended defects. In fact surfaces and grain boundaries share many similar features beyond their dimensionality such as modification of atom coordination and bonding, a strain field which decays with distance from the surface/grain boundary plane and a modified electronic structure compared to the bulk crystal. Indeed in many semiconducting materials the electronic properties of surfaces and grain boundaries are found to be closely related.^27^ Therefore, while modeling the properties of surfaces is of interest in their own right it can also provide an indication of the likely properties of grain boundary defects but with much reduced computational complexity, as demonstrated in our previous work on Sb_2_Se_3_.^14–16^ In this study, we model the structure and properties of surfaces in the pnictogen chalcohalide materials to provide insight into their electronic properties and by extension that of grain boundary defects. For each material we investigate the properties of the three lowest index surfaces. For all eight compounds we find that (following structural optimization) surfaces introduce only shallow electronic states near the band edges. The (100) surfaces cut covalent bonds in the 1D ribbons and initially introduce gap states, but some of these bonds are reformed following a facile reconstruction restoring bulk-like electronic structure. These results expand the set of chalcogenide materials that exhibit this novel effect and suggests further work on optimization with respect to point defects and heterointerfaces could deliver significant rewards in performance.

The space group of the eight chalcohalides we consider (BiSeI, BiSI, BiSeBr, BiSBr, SbSeI, SbSI, SbSeBr, SbSBr) is *Pnma* and all exhibit a very similar structure but with different lattice parameters. Figure 1a shows BiSeI, which we take as an exemplar material throughout this letter, with other materials found to be qualitatively similar (full details provided in the Supporting Information). Accurate prediction of the electronic properties of semiconductors typically requires either many-body perturbation theory or hybrid functionals (both with inclusion of spin–orbit coupling). The former is prohibitively expensive for large supercells such as those employed here. The latter is costly, but feasible if one can first obtain the structure using a less expensive exchange-correlation approximation. Unfortunately, there are known cases where employing this shortcut leads to inaccurate predictions.^28,29^ Therefore, it is essential to carefully validate the approach by comparing self-consistently optimized hybrid functional calculations of structure and band gap with the less expensive two-step approach. Table 1 summarizes the predicted lattice constants using both PBE+D3 and HSEsol+D3 functionals for all eight chalcohalides (see Methods). Geometry optimizations using PBE+D3 and HSEsol+D3 predict similar lattice constants, with the average percentage differences between the two functionals for a, b, and c being 1.9%, 2.6%, and 2.8%, respectively. Differences of more than 3% are only found for BiSI and SbSeI. This shows that while PBE+D3 is a far less computationally expensive functional choice, and is known to systemically underestimate band gaps, for structural optimization it is still a reasonable approximation.^30–33^ For the materials for which experimental structural data is available the predicted lattice parameters are in close agreement with the experimental results.^34–43^

**Figure 1:**
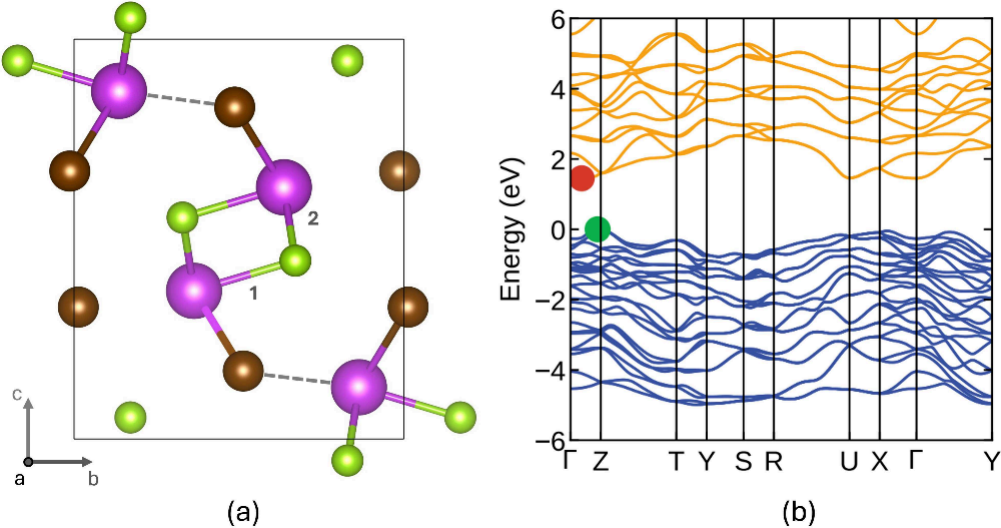
(a) Unit cell of BiSeI viewed in the [100] direction (Bi - purple, Se - green and I - brown). The dashed line highlights the Bi–I interchain bond, and (1) and (2) label the two inequivalent Bi–Se intrachain bonds. (b) Corresponding band structure calculated using HSEsol+D3+SOC and the PBE+D3 optimized geometry. Red and green points mark the conduction and valence band extrema.

**Table 1:** Predicted Band Gaps (*E*_g_) and Lattice Constants of Pnictogen Chalcohalides^a^

		PBE+D3 (Å)				HSEsol+D3 (Å)			
	Exp	DFT				DFT			
Material	*E*_g_ (eV)	*E*_g_ (eV)	*a*	*b*	*c*	*E*_g_ (eV)	*a*	*b*	*c*
SbSBr	1.8–2.2 ^21^	2.40	3.929	8.179	9.733	2.38	3.855	7.957	9.496
BiSBr	1.9 ^44^	2.06	4.058	8.150	9.784	2.20	3.984	7.940	9.508
SbSI	1.8–2.2 ^21^	1.98	4.056	8.430	10.119	1.98	3.986	8.217	9.817
SbSeBr	1.7 ^21^	1.87	3.999	8.271	10.224	1.90	3.916	8.041	9.971
SbSeI	1.8 ^20^	1.83	4.110	8.625	10.414	1.88	4.032	8.406	10.096
BiSI	1.6 ^45^	1.70	4.162	8.464	10.217	1.78	4.095	8.240	9.894
BiSeBr	1.5 ^46^	1.56	4.114	8.209	10.414	1.57	4.031	8.002	10.137
BiSeI	1.3 ^47^	1.45	4.217	8.667	10.522	1.53	4.120	8.430	10.220

a For each material optimized lattice parameters are obtained using both PBE+D3 and HSEsol+D3 approximations to exchange and correlation. The density functional theory (DFT) predicted band gap is calculated using the PBE+D3 structure and HSEsol+D3+SOC for the band structure calculation. Experimentally determined band gaps (exp) are also shown.

This MChX structure consists of 1D covalently bonded ribbons oriented along the [100] direction. In these materials one can distinguish two types of bond: strong intrachain bonds and weaker interchain bonds. The shortest interchain bonds are between M and X atoms (Figure 1a). The weaker interchain M–X bonds are longer than the corresponding intrachain M–X bonds by 14.7% for BiSeI and by 17.2% on average across all of the materials considered. Table S1 summarizes the lengths of these bonds for all eight chalcohalides. All atoms of the same species share the same coordination in the bulk structures, with pnictogens five coordinated, chalcogens three coordinated, and halogens two coordinated (note that here we define the coordination number in terms of the number of covalently bonded neighbors within the chains).

Table 1 also shows the experimental and calculated band gaps (using HSEsol+D3+SOC and the PBE+D3 structure) for each material. For comparison the band gaps are also calculated using the HSEsol+D3 optimized structure (using the same HSEsol+D3+SOC for band gap calculations). The two approaches are in very close agreement, with only one material having a difference in band gap of over 0.1 eV. This confirms that using PBE+D3 geometries and HSEsol+D3+SOC for optoelectronic properties is a reasonable approach to employ for surfaces (where the cost of full optimization using HSEsol+D3 would be too great). The predicted band gaps are also in good agreement with experimental data (Table 1). The band structure for BiSeI, calculated using this approach is shown in Figure 1b.

The bulk unit cells optimized using PBE+D3 are used to construct (100), (010) and (001) surface slab models for all eight chalcohalide materials. Surface formation energies are calculated both before and after structural optimization (Table 2). The atoms in the “before” configuration are positioned as they would be in a perfect bulk crystal. In the “after” configuration the atoms near the surface displace to adopt a lower energy structure. The difference in atom positions between the “before” and “after” configurations represents the strain field induced by the surface which diminishes with distance from the surface (the slab models should be sufficiently thick that the displacements in the center of the slab are very small, which we have verified is the case). The formation energy of the surface after structural optimization is the one relevant for predictions of surface stability but the change in the formation energy on structural optimization provides additional insight, with larger differences indicative of a more significant reconstruction, transforming a low stability termination into a more stable one.^48–50^ For all of the modeled materials the chalcogen-terminated (010) surfaces (see Figure S1 for BiSeI) are found to have the lowest formation energies. Intuitively, this is expected as creating the (010) surface is equivalent to cleaving the crystal between the 1D ribbons, and as such no intrachain bonds are broken. The most stable terminations of the (001) surfaces are halogen-terminated (see Figure S2 for BiSeI) and also do not break any intrachain bonds but involve a highly faceted surface, giving rise to a slightly higher surface energy. For both the (010) and (001) surfaces, the surface energy change during surface optimization is very low, with an average reduction of 12% for the (010) surfaces and 10% for the (001) surfaces. This is as expected, as no coordination changes occur for any of the atoms within the slabs during either surface formation, or surface relaxation. In contrast, forming the (100) surface necessities bisecting the 1D ribbon, and direct cleavage of a number of intraribbon bonds. Therefore, as one would expect the (100) surface formation energies before relaxation are much higher. However, during relaxation the surface energy decreases dramatically, falling by an average of 36%.

**Table 2:** Surface Formation Energies (*γ*) for Low Index Surfaces of Pnictogen Chalcohalide Materials^a^

	γ (J m^–2^)						*Δd*_M–X_^Inter^	
Material	(001)_*i*_	(001)_*f*_	(010)_*i*_	(010)_*f*_	(100)_*i*_	(100)_*f*_	(%)	*Δγ*_(100)_
SbSBr	0.33	0.31	0.28	0.25	0.74	0.42	–8.8	0.32
BiSBr	0.41	0.35	0.37	0.30	0.80	0.52	–9.6	0.28
SbSI	0.33	0.31	0.27	0.25	0.68	0.41	–7.9	0.27
SbSeBr	0.33	0.30	0.27	0.24	0.70	0.42	–10.6	0.28
SbSeI	0.33	0.32	0.27	0.25	0.66	0.42	–9.0	0.24
BiSI	0.37	0.33	0.31	0.28	0.68	0.48	–8.5	0.20
BiSeBr	0.39	0.33	0.32	0.27	0.75	0.49	–12.0	0.26
BiSeI	0.37	0.33	0.30	0.26	0.69	0.47	–9.7	0.22

a Formation energies are given for the initial surface (as cleaved from the bulk crystal) and the fully-optimized surface indicated by *i* and *f* subscripts respectively. For the (100) surface the change in length of the inter-chain M–X bond length following relaxation (*Δd*_M–X_^Inter^) and the change in formation energy (*Δγ*_(100)_) are also given.

To provide further insight into the relaxation of the (100) surface the structure for BiSeI is shown in Figure 2 (with the nature of the relaxation qualitatively similar for all the other materials, see Figures S3–S9). For analysis of the structural changes it is helpful to define a distance below which we consider a strong bond to have formed. For this purpose we take the average of the longest intrachain bond and the shortest interchain bond of each type as a cutoff distance. This results in a cutoff distance for the Bi–I bonds in BiSeI of 3.49 Å. Before relaxation, the coordination numbers of atoms in the surface layer (L1 in Figure 2) is reduced: pnictogens from five to three (losing a bond to both a chalcogen and halogen atom), chalcogens from three to two (losing a bond to a pnictogen), and halogens from two to one (losing a bond to a pnictogen). Following relaxation, halogens in the surface layer (L1) form new interchain bonds to pnictogens in the subsurface layer (L2). These new bonds are highlighted in Figure 2 and Table 2 summarizes the change of the interchain M–X bond length following relaxation for all materials. While this reconstruction still leaves pnictogen and chalcogens in the surface layer uncoordinated, it restores the coordination of the surface halogen to two (the same as in the bulk crystal). It also increases the coordination of pnictogens in the subsurface layer from five to six. The main contribution to the reduction of *d*_Bi–I_^Inter^ is the outward relaxation of pnictogens in the subsurface layer (L2).

**Figure 2:**
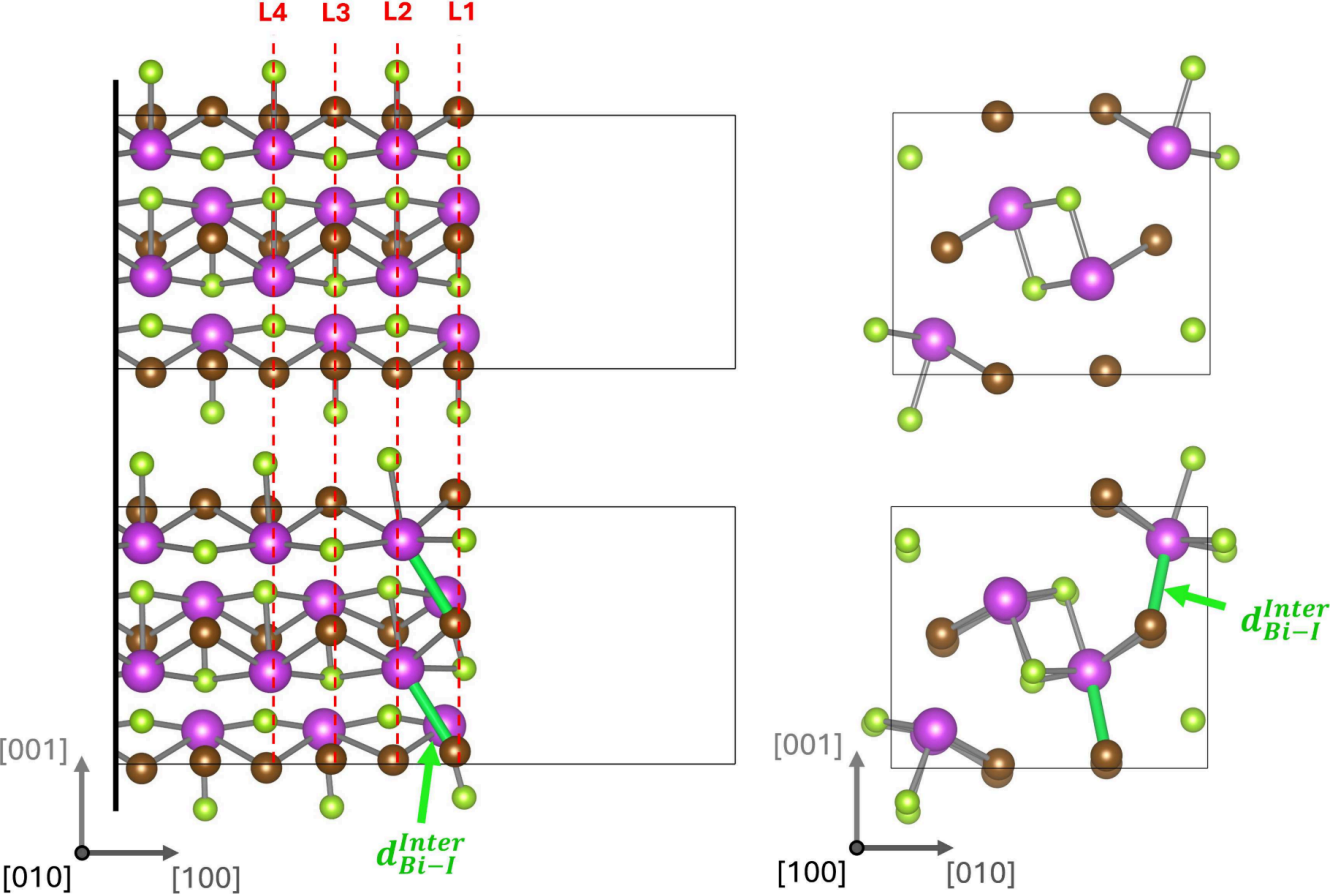
(100) surface of BiSeI before (top row) and after (bottom row) structural optimization. Projections are shown in the [010] (left column) and [100] (right column) directions. For the [010] projection the dashed lines (labeled L1–L4) indicate the positions of atomic planes in the structure before relaxation. For the [010] projection, only the outermost six layers (L1–L6) of atoms from one side of the slab are shown. For the [100] projection only the outermost three layers (L1–L3) of atoms are shown. The newly formed inter-ribbon Bi–I bond with length *d*_Bi–I_^Inter^ is highlighted in green.

The spacing between the outermost layers of atoms (L1 and L2) decreases in all of the surfaces with BiSeI experiencing an interlayer contraction of 4.8%. In contrast the spacing between atoms in L2 and L3 is expanded by 6.0%. This oscillatory strain continues deeper into the slab with decreasing amplitude: L3–L4 spacing −1.2%, L4–L5 spacing +0.8%. This can be understood as a result of the formation of new interchain bonds at the surface which leads to a tighter bonding between the two outermost surface layers and an oscillatory strain field that propagates below the surface. All other chalcohalide materials exhibit very similar effects (see Figures S3–S9 and Table S2).

To analyze the electronic structure of the surfaces we compute the density states (DOS) for the slabs in the unrelaxed bulk-like (“before”) and relaxed (“after”) configurations and project the densities of states in both near surface and bulk regions (i.e., the center of the slab). Analogous to the analysis of structure and stability presented above, comparing the electronic structure for these different configurations provides additional insight beyond computation of the relaxed structure alone. For example, for a sufficiently thick slab (such that the strain in the central bulk region is small) the DOS projected in the bulk region should be very similar before and after relaxation and equivalent to that of a 3D periodic bulk crystal. For all the calculations we have performed we have verified this is the case. A DOS projection in the surface region for the (001) and (010) surfaces reveals that these surfaces do not generate any gap states, either before, or after, relaxation (see Figure S10 and S11 for BiSeI). However, the behavior of the (100) surface is very different as shown in Figure 3a for BiSeI. The projected density of states in the surface region (L1–L2) shows that before relaxation a gap state is formed via the cleavage of bonds at the surface. However, after relaxation we can see that this gap state disappears; i.e., the reconstruction has eliminated (or self-healed) the gap state. This same effect is observed in all of the materials investigated (see Figures S12–S18). It shows that the specific reconstruction of this surface is essential for eliminating the gap state, which is not a conclusion one could make on the basis of analyzing the “after” configuration only. Before relaxation, the projected density of states in the bulk region (L7–L10) shows no gap states, or evidence of other effects impacting the electronic structure at the center of the slab. After relaxation, this remains largely unchanged, with a slight distortion indicating that the strain generated from changes to bond lengths at the surface has a small impact in this central region.

**Figure 3:**
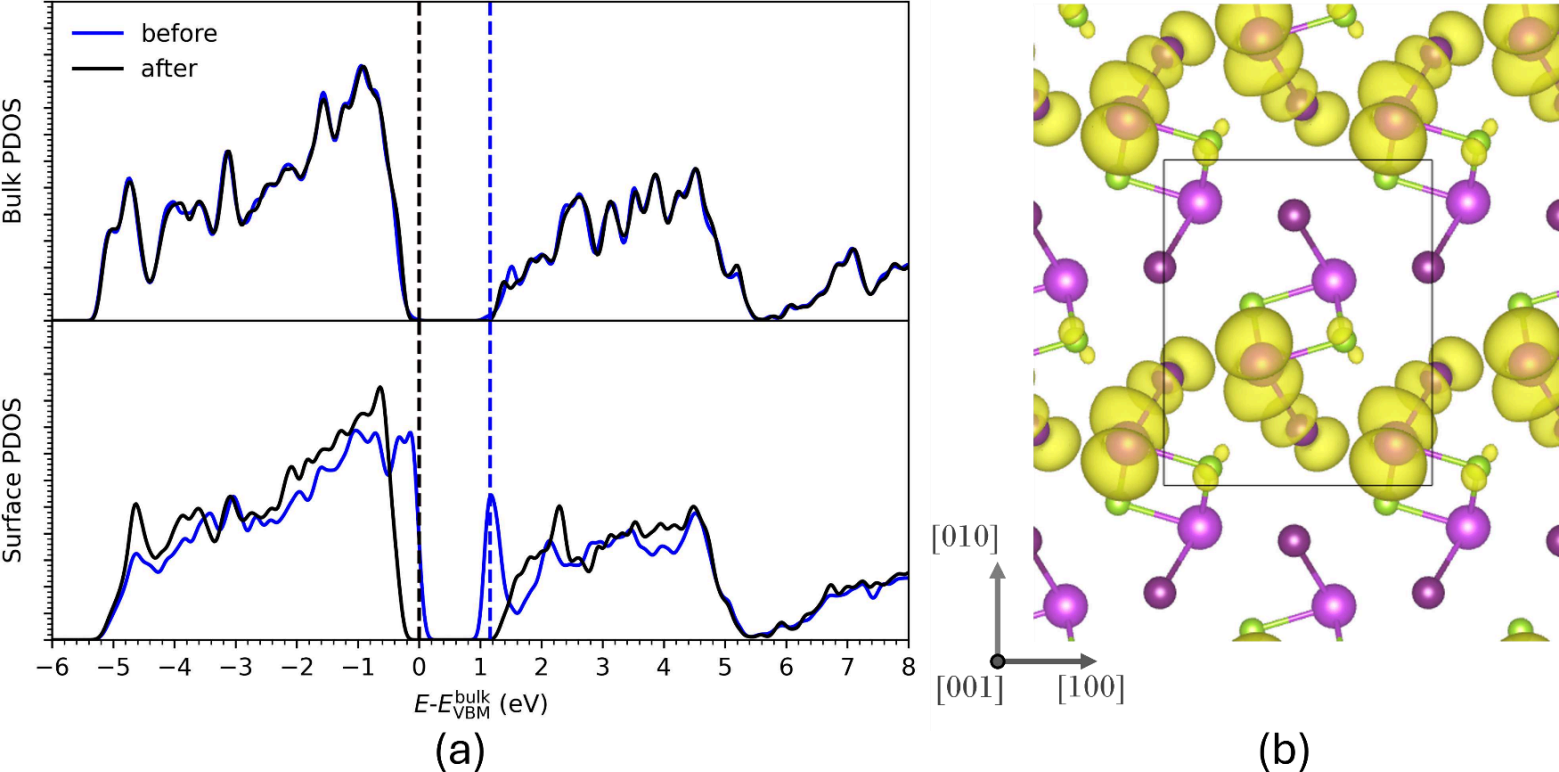
(a) Density of states data for BiSeI, showing the projected density of states in the surface and bulk regions of the slab, both before and after relaxation. The dashed lines indicate the positions of the band edges for the bulk region. (b) A visualization of charge density for the gap states at the surface before relaxation viewed along the [001] direction.

To provide further insight into the nature of the gap states in the unreconstructed (100) surface, we compute and visualize charge densities associated with the specific bands in the gap at the Γ point (Figure 3b). We find that charge is predominantly localized around three distinct atoms: the pnictogen and chalcogen atoms at the surface (L1) and the halide atom just beneath the surface (L2). The two surface atoms have reduced coordination compared to the bulk. The subsurface halide maintains its bulk-like coordination. However, during relaxation, we note that the bond between this halide and the surface pnictogen is very significantly reduced in length, by around 8.8%—the largest contraction of any pre-existing bond within the structure. Calculation of atom projected DOS at the surface also confirms the gap state has contributions from the pnictogen atom, the halide atom and the chalcogen atom, in order of decreasing contribution (see Figure S19).

The predicted reconstruction behavior observed in the pnictogen chalcohalides shows notable parallels to the previously studied Sb_2_S/Se_3_, particularly in the mechanism of gap state healing via the formation of new interchain bonds.^14^ However, a significant difference emerges in the coordination changes accompanying reconstruction. Whereas the reconstruction in Sb_2_S/Se_3_ leads to restoration of bulk-like coordination, the pnictogen chalcohalides retain a nonbulk-like coordination even after reconstruction. The pnictogen and chalcogen atoms in the topmost surface layer (L1) remain undercoordinated, with coordination numbers decreasing from five to three and three to two, respectively. Additionally, the pnictogen atoms in the second layer (L2) become overcoordinated, increasing from five to six. Nevertheless, the end result in terms of eliminating gap states is similar, suggesting these specific undercoordinated atoms in chalcohalides do not induce gap states. We note we cannot rule out more complex longer period reconstructions which could be more stable than those we predict. However, in structures obtained by simple energy minimization the dangling bond gap states are eliminated and the resulting surface energies are low, providing limited impetus for more complex reconstructions.

Direct experimental validation of the predicted surface reconstructions is challenging. For example, atomic-resolution scanning probe microscopes are limited by their requirement for atomically flat surfaces and their inability to directly probe subsurface layers, which are crucial in these reconstructions. However, with suitable samples this may be possible in the future as has been demonstrated for other novel solar cell materials, such as organic and halide perovskite, solar cells.^51,52^ While direct experimental confirmation of these reconstructions remains difficult, indirect evidence, such as the long-range strain fields associated with the reconstruction, may be observed via techniques such as scanning transmission electron microscopy as recently demonstrated for Sb_2_Se_3_.^16^ We also note that the present study addresses ideal chalcohalide surfaces exposed to a vacuum in order to understand the intrinsic properties of such extended defects. When surfaces are exposed to an environment, such as in photoelectrochemical cells, surface composition and structure can be affected by interaction with various chemical species (e.g., from solutions or the atmosphere) which would require extension on the models presented here.^53–56^

The predicted surface electronic structure of the pnictogen chalcohalides is notably different from the previously studied and structurally similar semiconductors Sb_2_S_3_ and Sb_2_Se_3_. Even prior to reconstruction, these materials exhibit much shallower surface states (typically between 200 and 500 meV below the conduction band minimum) suggesting a reduced tendency for formation of deep gap states.^14^ While Sb_2_S/Se_3_ undergoes a more complete surface reconstruction, mitigating its deeper defect states, pnictogen chalcohalides retain residual band edge distortions after relaxation. However, given the initial shallowness of the surface states, this residual undercoordination has a less pronounced electronic impact. Consequently, even if full reconstruction is not achieved, the negative impact of extended defects is expected to be relatively limited compared to materials like CdTe, where deeper surface or interface states have been shown to form.^57^

While experimental detection of shallow gap states remains experimentally challenging, spectroscopic measurements may offer indirect evidence of such states. Similar approaches have been successfully used in related semiconductors, such as CZTS, to quantify defect-related band edge effects.^58,59^ For quantitative comparison one would need to go beyond the hybrid DFT calculations presented here and employ many-body approaches such as the GW approximation and the Bethe–Salpeter Equation which can provide predictive accuracy for excitonic absorption features.^60,61^

While this study has focused on predicting the atomic structure and electronic properties of surface extended defects, it is likely many of the general conclusions will also be applicable to grain boundary defects. This is supported by prior studies on materials such as anatase TiO_2_, MgO, and Sb_2_S/Se_3_ which exhibit similar electronic properties at both surfaces and internal interfaces.^27,62,63^ However, to confirm this directly for chalcohalides future work should focus on modeling grain boundary defects, with complementary information provided by techniques such as scanning transmission electron microscopy to validate models.^27^

The predicted reconstruction and shallow gap states of the pnictogen chalcohalides may offer a substantial advantage over other thin-film semiconductors. For example, in CdTe grain boundaries often introduce deep electronic states that enhance nonradiative recombination, necessitating postdeposition treatments to passivate these defects.^64,65^ In contrast, the intrinsic ability of pnictogen chalcohalides to reconstruct and eliminate deep gap states at extended defects implies a lower level of defect-related recombination. However, despite their apparent tolerance to extended defects, point defects remain a limiting factor. Both theoretical and experimental work on related chalcohalides suggests that intrinsic defects, particularly vacancies, can introduce localized states within the band gap, thereby limiting achievable device efficiency.^25^ Therefore, defect segregation to grain boundaries remains a potential concern. In other semiconductor systems, such as CdTe and CIGS, such segregation leads to local band bending, space-charge effects, and carrier trapping - all of which can be detrimental to photovoltaic performance.^66–68^ Reports on thin-film devices based on chalcohalides demonstrate efficiencies in the range of 4–6%, underscoring the need to better control defects in order to improve the potential of these systems.^69^

Several promising avenues exist for further investigation of extended defects in pnictogen chalcohalide. First, while current predictions are based on vacuum conditions, surface reconstructions are likely to differ under realistic environments, such as in the presence of water, oxygen, or during photoelectrochemical operation. Future work could therefore include simulations to capture the impact of environmental conditions on surface structure and stability.^70,71^ The development of machine learning interatomic potentials offers a powerful tool for high-throughput screening of materials with favorable properties; specifically, those capable of reconstructing without forming dangling bonds. This could enable accelerated discovery of defect-tolerant materials within this family and beyond. Additionally, explicit modeling of grain boundaries, coupled with complementary investigation via transmission electron microscopy techniques, could provide deeper insight into how surface-derived predictions translate to internal interfaces.^72–74^ Another important issue to address is the interaction between point and extended defects. While point and extended defects separately may not contribute significantly to nonradiative recombination in the bulk, segregation of point defects to grain boundaries could increase recombination activity. Understanding these interactions will be essential for optimizing device performance. Finally, direct calculation of nonradiative recombination rates, especially using first-principles methods or hybrid-functional approaches, is key to gaining a full understating of the impact of defects on device efficiency.^63,75–77^

To summarize, we have investigated the structure and properties of extended defects in eight pnictogen chalcohalide materials to explore their potential resistance to the formation of electronic states in their band gaps with relevance to applications in photovoltaics and optoelectronics. We find that even when extended defects disrupt covalent bonding, exposing dangling bonds with associated gap states, a facile reconstruction leads to the elimination of electronic states from the band gap region. The reconstruction involves formation of new interchain bonds at the surface which is accommodated by the subsurface layers and induces significant strain. This gap-state healing behavior is very unusual, having only recently been predicted for the structurally similar materials Sb_2_Se_3_ and Sb_2_S_3_,^14,15^ and suggests the electronic structure of this wider family of materials has an intrinsic tolerance to extended defects. This positions pnictogen chalcohalides as promising candidates for polycrystalline thin-film photovoltaics.

## Methods

Spin-polarized density functional theory (DFT) calculations using a plane wave basis set and the projector augmented wave (PAW) method were carried out using the Vienna Ab Initio Simulation Package (vasp 6.5.1).^78,79^ Bulk structures and properties of the materials were first obtained using the PBE (Perdew–Burke–Ernzerhof) exchange-correlation functional^80^ and PAW PBE pseudopotentials from the VASP 5.4 library with the following electrons considered as valence: Bi (5d^10^6s^2^6p^3^), Sb (4d^10^5s^2^5p^3^), S (3s^2^3p^4^), Se, (4s^2^4p^4^) and I (5s^2^5p^5^). Structural optimizations employed a 6 × 3 × 2 Γ centered Monkhorst–Pack k-point grid and a 500 eV cutoff for the plane-wave basis set without consideration of spin–orbit coupling. The bulk structures were optimized to a force tolerance of 0.005 eV/Å. We also employed the HSEsol (Heyd–Scuseria–Ernzerhof for solids) functional,^81^ which includes nonlocal exchange for more accurate modeling of the electronic structure of the materials under study.^81^ To account for van der Waals interactions between the ribbons which are otherwise poorly represented by PBE and HSEsol we included D3 Grimme dispersion corrections with Becke-Johnson damping, utilizing parameters taken from the Simple DFT-D3 library (hereafter referred to as PBE+D3 and HSEsol+D3).^82,83^ The values used for the van der Waals damping parameters for HSEsol are *a*_1_ = 0.4650, *a*_2_ = 6.2003, and *s*_8_ = 2.9215. While not employed for structural optimizations, spin–orbit coupling is included for the electronic calculations calculations performed on the bulk structures (hereafter HSEsol+D3+SOC).

Surfaces were modeled using the slab approach, where an appropriately oriented unit cell (optimized using the PBE+D3 functional, and the calculation parameters detailed above) is repeated in the direction of the surface normal, truncated and a vacuum gap added to generate a two-dimensional slab of finite thickness.^84–86^ We tested varying slab thicknesses, from 10 Å to 50 Å to determine the optimal number of layers such that center of the slab retains a bulk-like structure following optimization of the surface slab. We found that a slab thickness of at least 30 Å, corresponding to eight unit cells for the (100) surface, was sufficient for these surfaces with only a 0.6% extension of the M–X bonds in the center of the slab compared to the bulk. Similarly, vacuum gap thicknesses of 10 Å to 25 Å were tested, and a vacuum gap of 15 Å was found to be sufficient to minimize interactions between the periodically repeated slabs. The structure of the surfaces were optimized (with supercell volume fixed) using PBE+D3, an energy cutoff of 500 eV, a force tolerance of 0.005 eV/Å and without inclusion of spin–orbit coupling. For the structural optimizations of these surfaces, a Monkhorst–Pack *k* point grid of 6 × 3 × 1 was chosen for (001) surfaces, 6 × 2 × 1 for the (010) surfaces, and 3 × 2 × 1 for the (100) surfaces.

The formation energies of the surfaces (γ) before and after optimization were calculated using the following equation,

1 $$\gamma =\frac{E_{\mathrm{slab}}-NE_{\mathrm{bulk}}}{2A}$$

where *E*_slab_ is the total energy of the slab, *N* is the total number of formula units within the slab, *E*_bulk_ is the bulk total energy per formula unit and *A* is the total surface area of one side of the slab. *E*_bulk_ is obtained by calculating the total energy per formula unit of a relaxed bulk unit cell using equivalent parameters to the surface calculation.

The density of states of the surfaces before and after optimization were computed using HSEsol+D3+SOC (with 25% Hartree–Fock exchange) using the PBE+D3 optimized structures. For the HSEsol+D3+SOC calculations the k-point grids used were 6 × 2 × 1 for (001) surfaces, 6 × 2 × 1 for the (010) surfaces, and 4 × 2 × 1 for the (100) surfaces. These slightly modified grid sizes were chosen to be divisible by two to allow for down-sampling of the exact exchange contributions on smaller grids of 3 × 1 × 1 for (001) surfaces, 3 × 1 × 1 for the (010) surfaces, and 2 × 1 × 1 for the (100) surfaces (using the NKRED option in VASP). The HSEsol+D3+SOC calculations were initialized with wave functions obtained using PBE+D3+SOC. So the full sequence of calculations is optimization using PBE+D3, a single point calculation using PBE+D3+SOC and a single point using HSEsol+D3+SOC (where the exact exchange contributions are evaluated on a coarser k-point grid). Using this technique, the HSEsol+D3+SOC calculations are approximately ten times more expensive as the PBE+D3+SOC ones. The densities of states projected onto bulk and surface regions were also computed and band-projected charge densities were obtained to visualize the spatial localization of surface states within the band gap.

The structures and band-projected charge densities were visualized using the vesta software package.^87^ The surfaxe package was utilized for generating the surface slab supercells, as well as for determining the appropriate slab thickness and the vacuum gap between periodic images.^88^ The sumo python toolkit was used for density of states projections and matplotlib used to generate plots comparing surface and bulk projected densities of states before and after optimization.^89,90^

## Supplementary Material





## Data Availability

All data created during this research are available by request from the University of York Research database (https://doi.org/10.15124/53b311d8-f063-47c6-b78f-1a2af9cfd619).

## Notes

A table summarizing intra- and interchain bond lengths in the pnictogen chalcohalide materials. Structures of the (010) and (001) surfaces of BiSeI and (100) surfaces for all materials excluding BiSeI (before and after relaxation). A table showing strains induced by the (100) surface reconstruction in each material. Projected density of states for each of the (100) surfaces (before and after relaxation). Atom projected density of states for the (100) surface of BiSeI (PDF)
Transparent Peer Review report available (PDF)
