# Supplementary material for: Structural and Electronic Reconstruction of Extended Defects in Pnictogen Chalcohalides
Source: J Phys Chem Lett. 2026 Mar 4;17(11):3142–51. doi: 10.1021/acs.jpclett.5c03107 (PMC13007012; doi:10.1021/acs.jpclett.5c03107)
Supplement: Supplementary file 1 [file jz5c03107_si_001.pdf]

Supporting Information: Structural and Electronic Reconstruction of Extended Defects in Pnictogen Chalcohalides

Thomas Lynch,*,† Cibrán López,‡,¶ Claudio Cazorla,‡,¶ and Keith P. McKenna*,†

†*School of Physics, Engineering and Technology, University of York, York, YO10 5DD, UK*

‡*Departament de Física, Universitat Politècnica de Catalunya, 08034 Barcelona, Spain*

¶*Barcelona Research Center in Multiscale Science and Engineering, Universitat Politècnica de Catalunya, 08019 Barcelona, Spain*

E-mail: tom.lynch@york.ac.uk; keith.mckenna@york.ac.uk

Table S1: Intra- and inter-chain bond lengths for chalcohalides obtained using PBE+D3. See Fig. 1a for the definition of the two inequivalent M-X intra-chain bonds. All distances given in Å.

	$d^{\text{Intra}}_{\text{M-Ch,1}}$	$d^{\text{Intra}}_{\text{M-Ch,2}}$	$d^{\text{Intra}}_{\text{M-X}}$	$d^{\text{Inter}}_{\text{M-X}}$
SbSBr	2.51	2.66	2.94	3.56
BiSBr	2.62	2.71	3.03	3.47
SbSI	2.51	2.69	3.12	3.71
SbSeBr	2.66	2.78	2.98	3.59
SbSeI	2.65	2.80	3.15	3.75
BiSI	2.62	2.75	3.22	3.68
BiSeBr	2.76	2.84	3.07	3.53
BiSeI	2.75	2.86	3.25	3.73

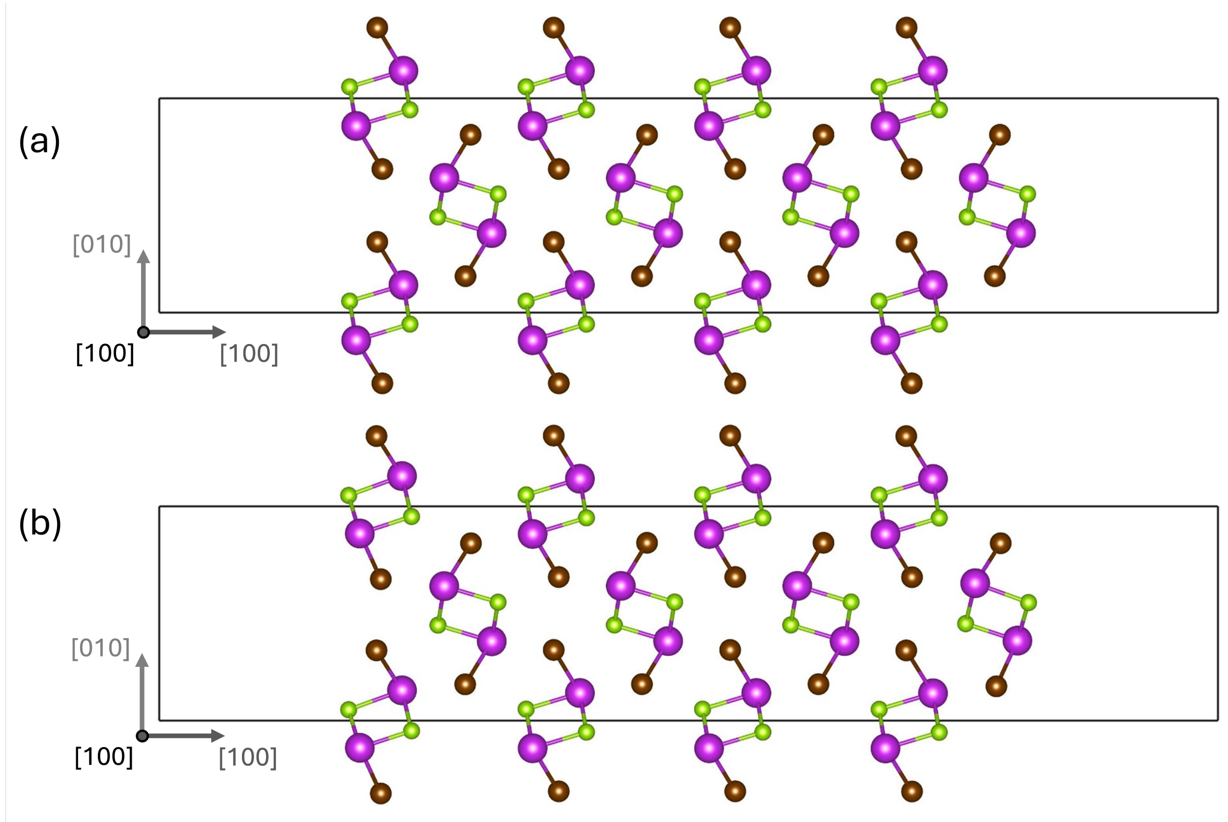

Figure S1: The (010) surface of BiSeI before (a) and after (b) structural optimization. Projections are shown in the [001] direction. Note that the image has been expanded to show structural ribbons which overlap the boundary of the computational cell.

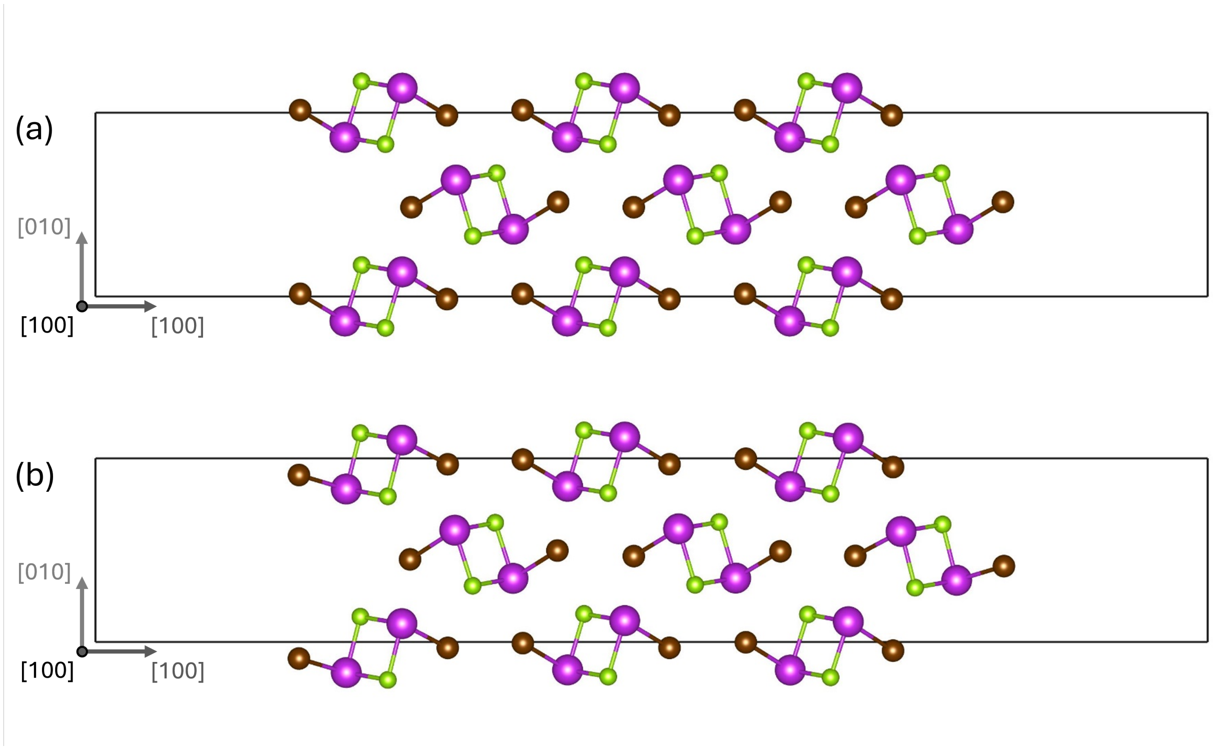

Figure S2: The (001) surface of BiSeI before (a) and after (b) structural optimization. Projections are shown in the [001] direction. Note that the image has been expanded to show structural ribbons which overlap the boundary of the computational cell.

Table S2: Percentage difference from bulk interplanar distances, for the first 8 layers, for every pnictogen chalcohalide investigated.

	Percentage difference from bulk (Å)						
	L1-L2	L2-L3	L3-L4	L4-L5	L5-L6	L6-L7	L7-L8
BiSBr	-6.1	7.5	-1.8	1.4	-0.9	0.5	-0.6
BiSeBr	-6.5	8.4	-1.7	1.5	-0.5	0.6	-0.1
BiSeI	-4.8	6.0	-1.2	0.8	-0.4	0.0	-0.1
BiSI	-5.2	6.3	-1.7	1.2	-0.6	0.4	-0.1
SbSBr	-7.3	11.1	-3.6	3.6	-2.0	2.0	-1.2
SbSeBr	-7.2	10.4	-2.9	2.5	-1.3	1.3	-0.8
SbSeI	-5.5	7.2	-2.3	1.6	-1.1	0.7	-0.6
SbSI	-6.2	8.7	-3.4	2.7	-1.7	1.5	-1.0
Average	-6.1	8.2	-2.3	1.9	-1.1	0.9	-0.6

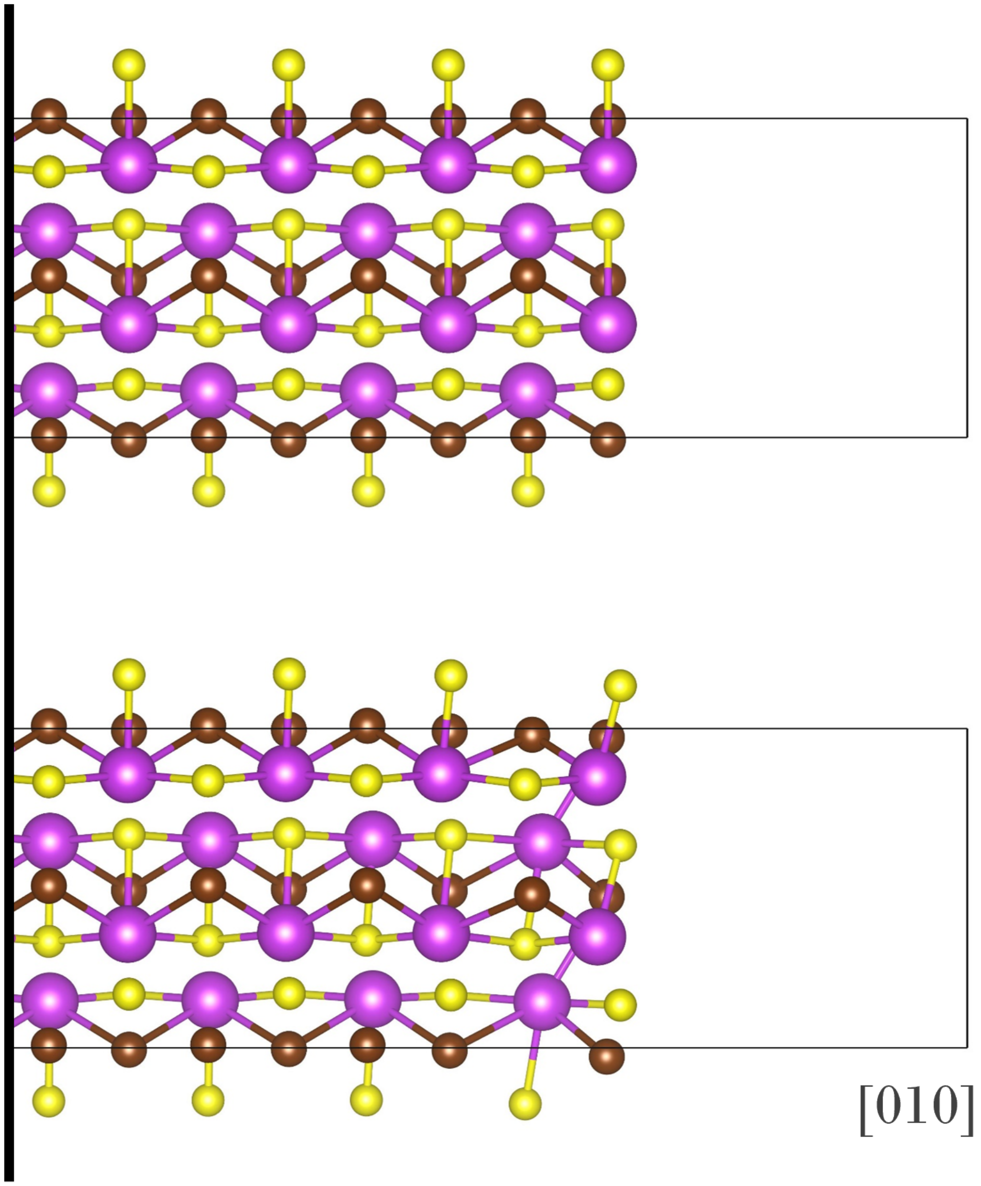

Figure S3: The (100) surface of BiSBr before (top row) and after (bottom row) structural optimization. Projections are shown in the [010] direction

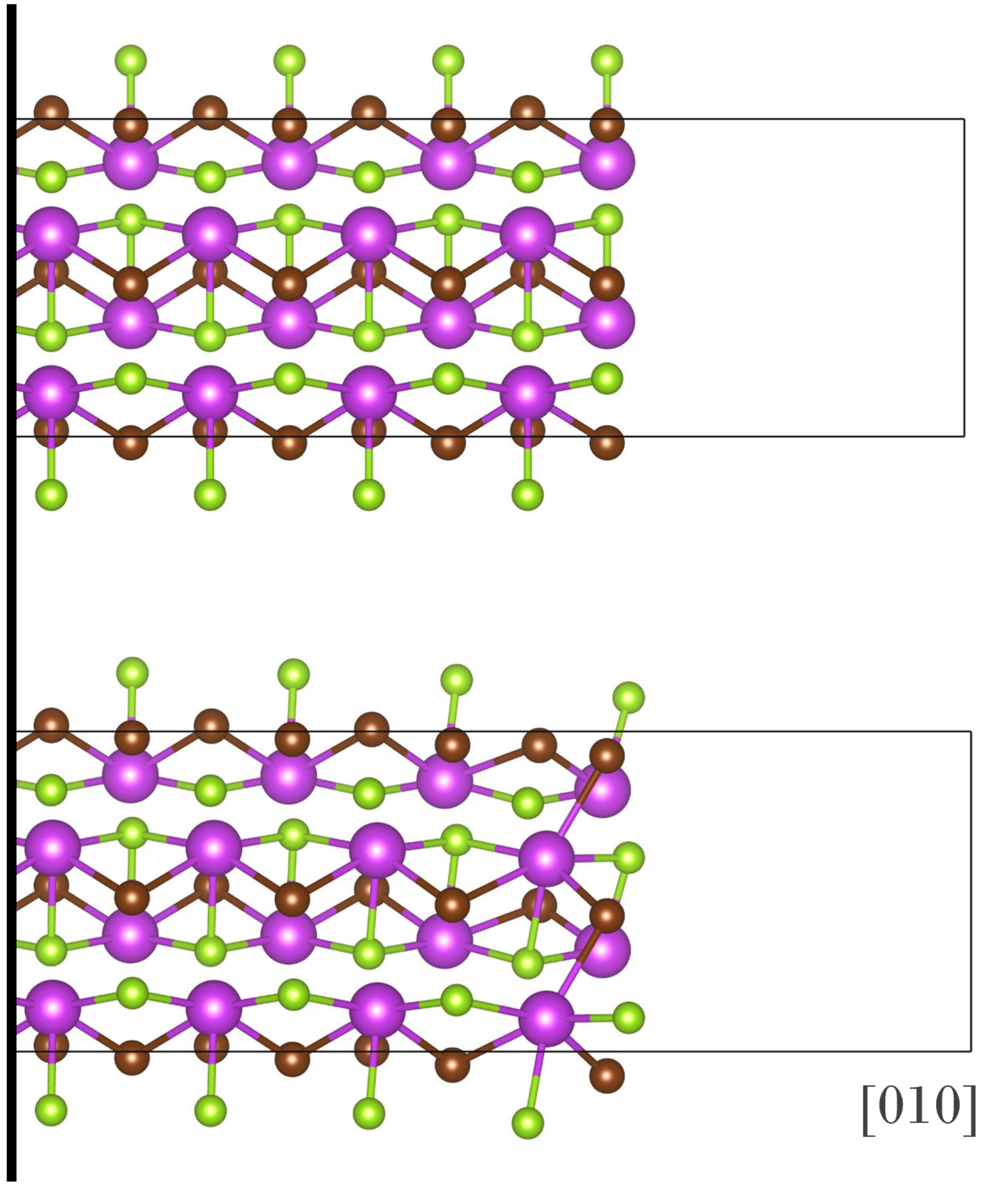

Figure S4: The (100) surface of BiSeBr before (top row) and after (bottom row) structural optimization. Projections are shown in the [010] direction

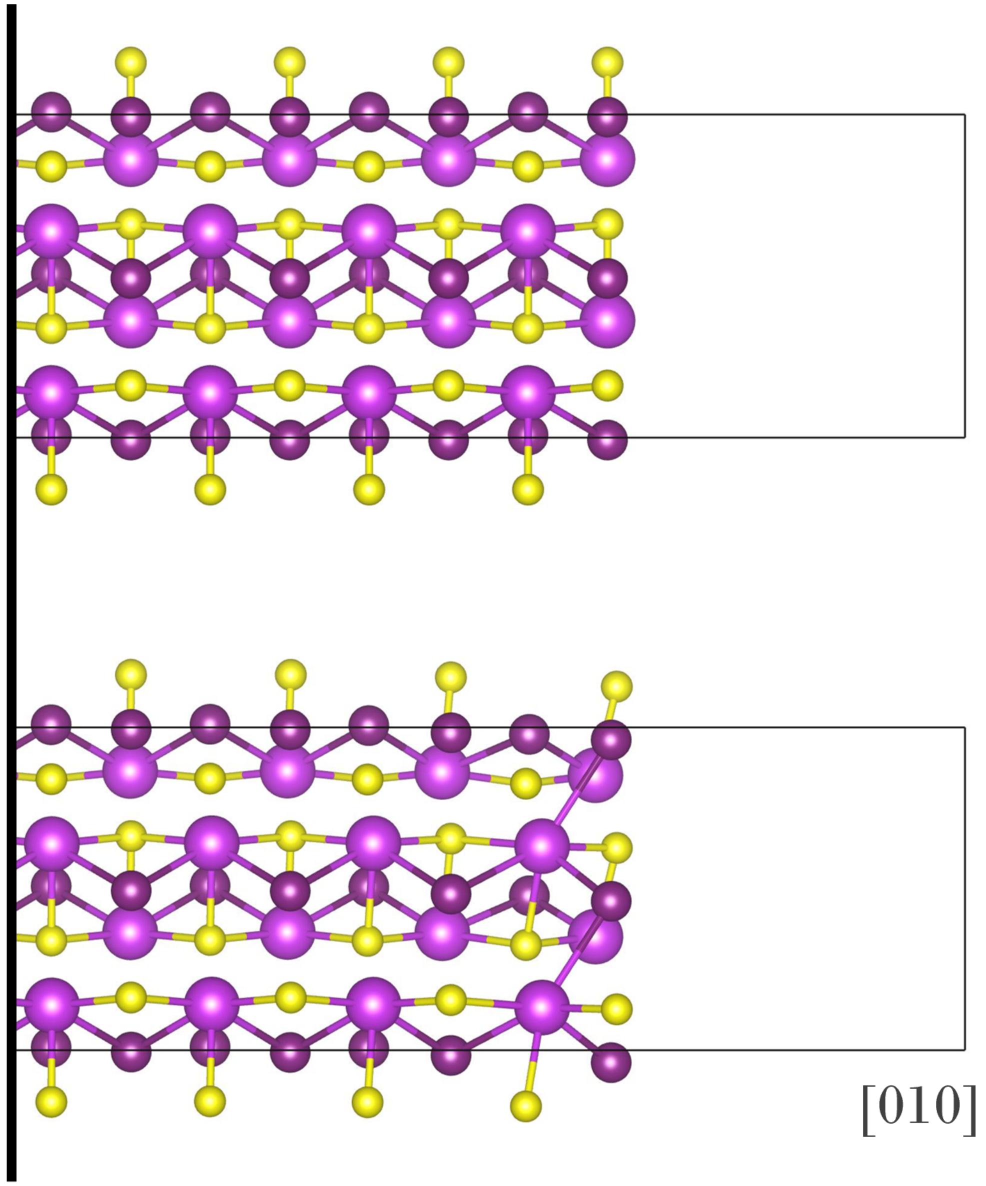

Figure S5: The (100) surface of BiSI before (top row) and after (bottom row) structural optimization. Projections are shown in the [010] direction.

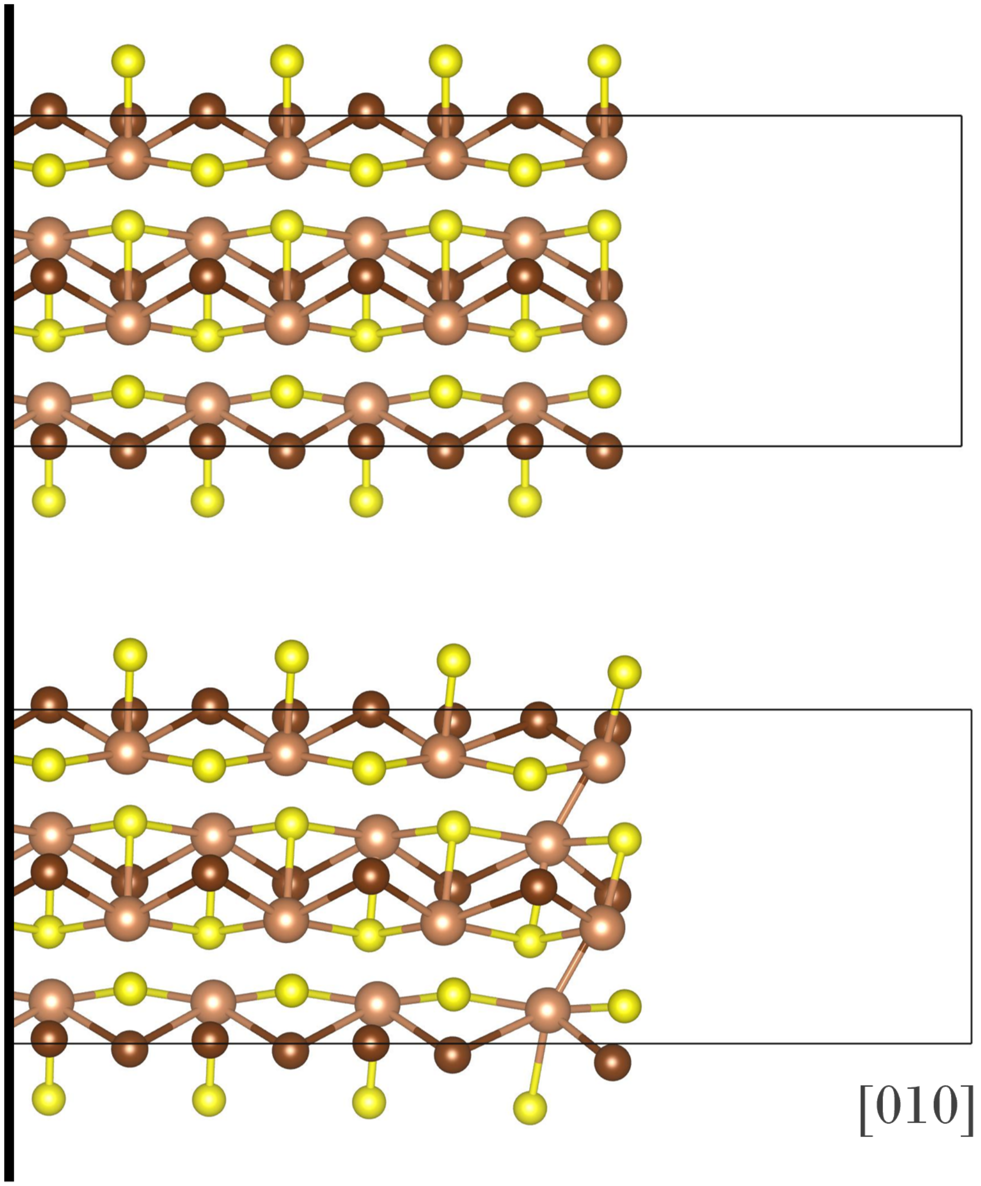

Figure S6: The (100) surface of SbSBr before (top row) and after (bottom row) structural optimization. Projections are shown in the [010] direction.

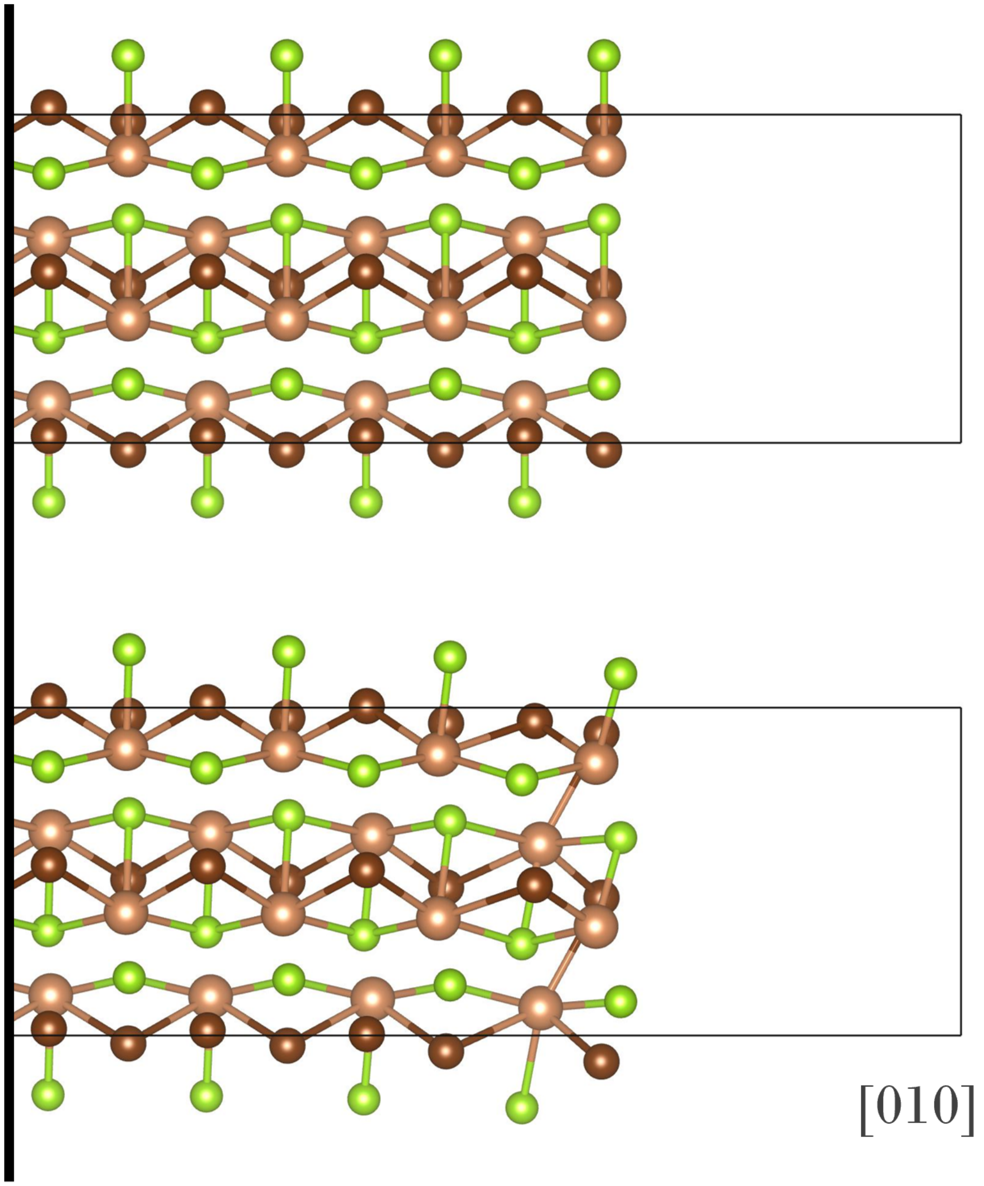

Figure S7: The (100) surface of SbSeBr before (top row) and after (bottom row) structural optimization. Projections are shown in the [010] direction.

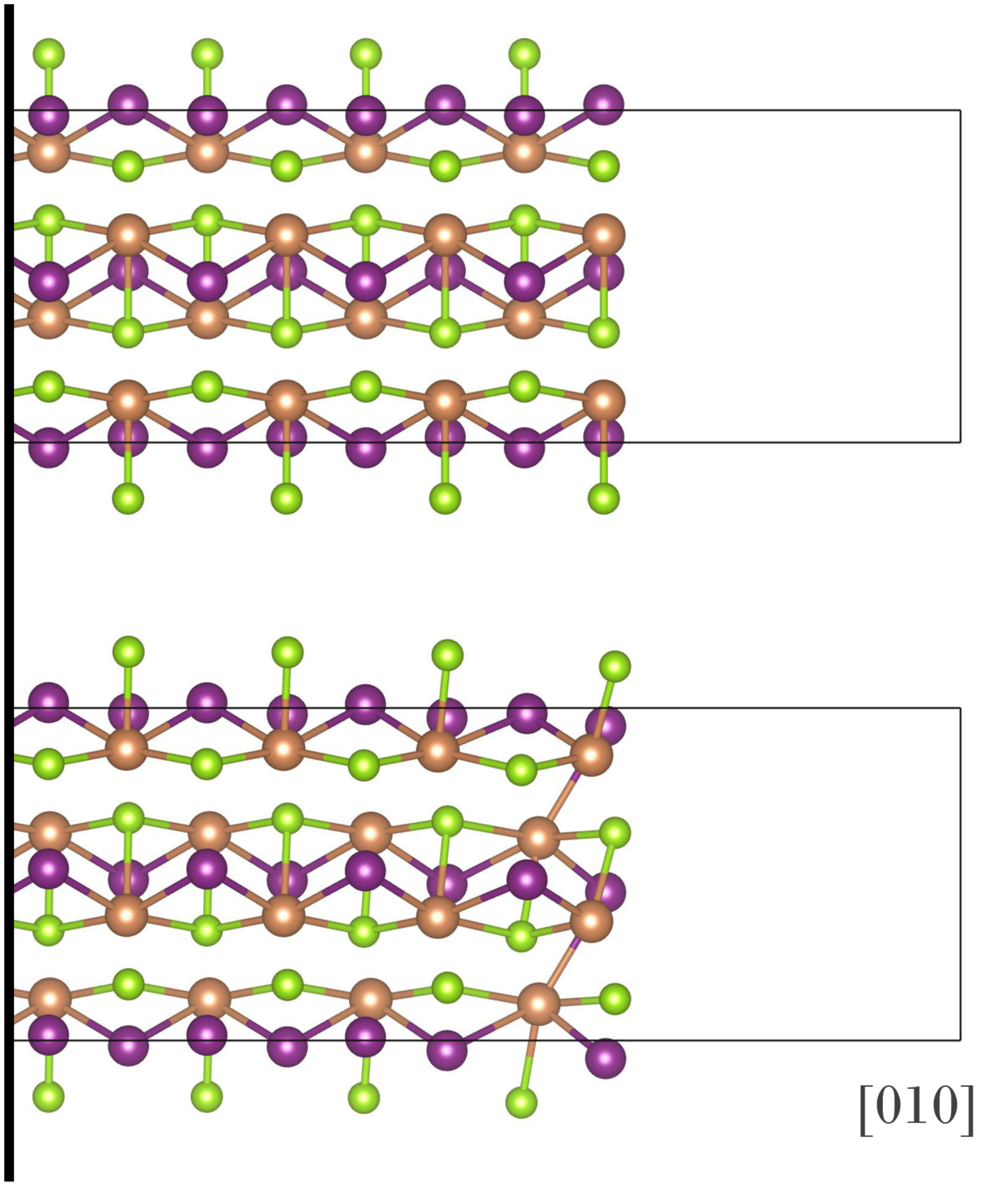

Figure S8: The (100) surface of SbSeI after structural optimization. Projections are shown in the [010] direction.

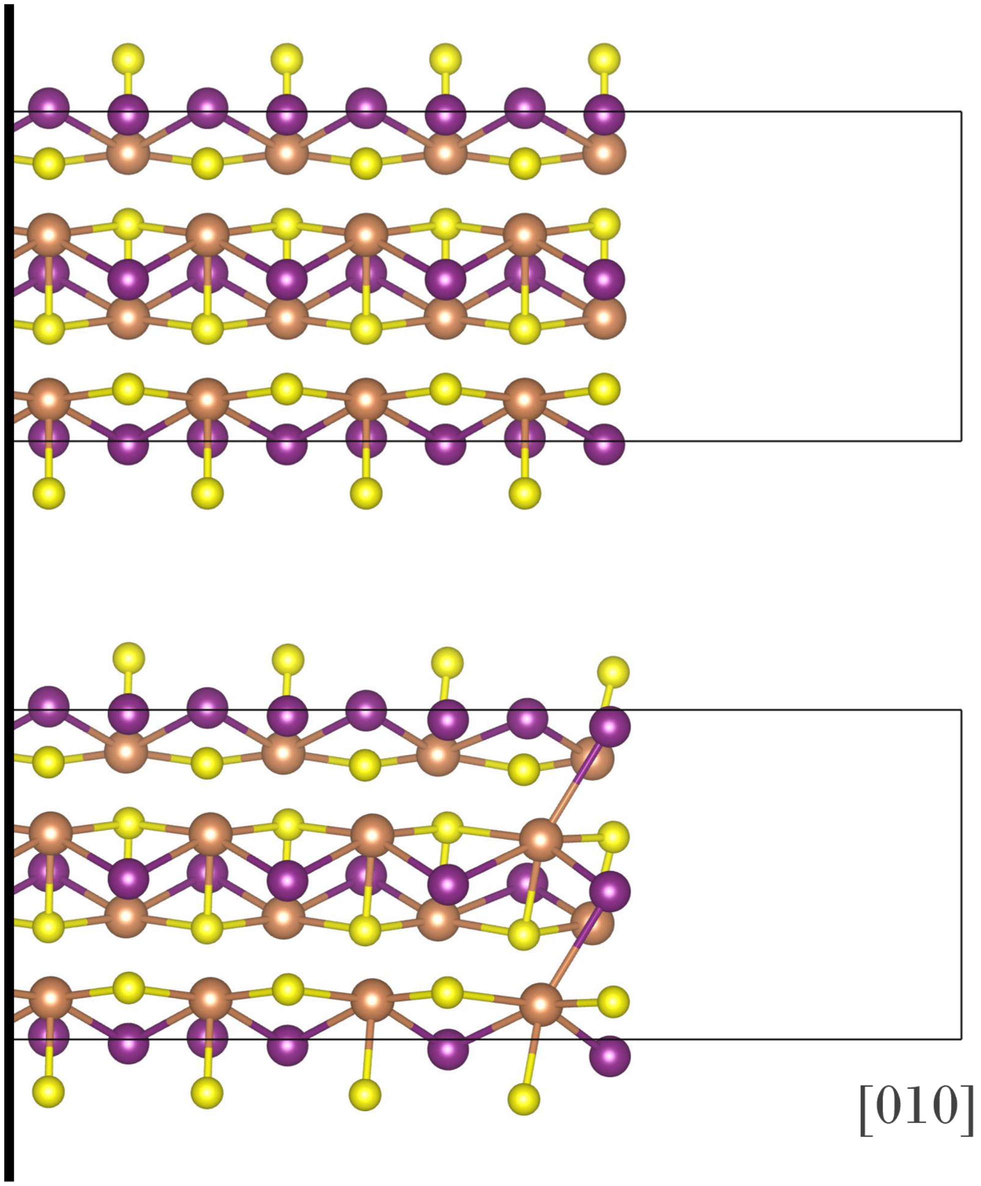

Figure S9: The (100) surface of SbSI after structural optimization. Projections are shown in the [010] direction.

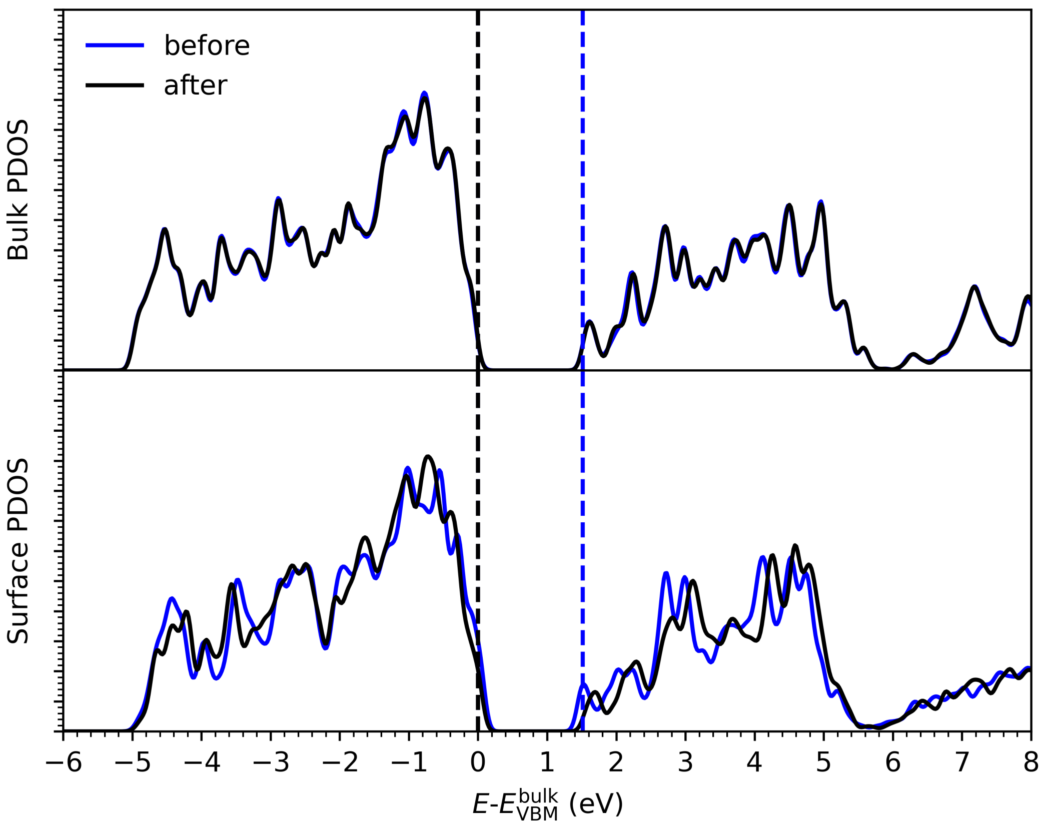

Figure S10: Density of states data for the (010) surface of BiSeI, showing PDOS taken at the surface and centre of the slab, both before and after relaxation.

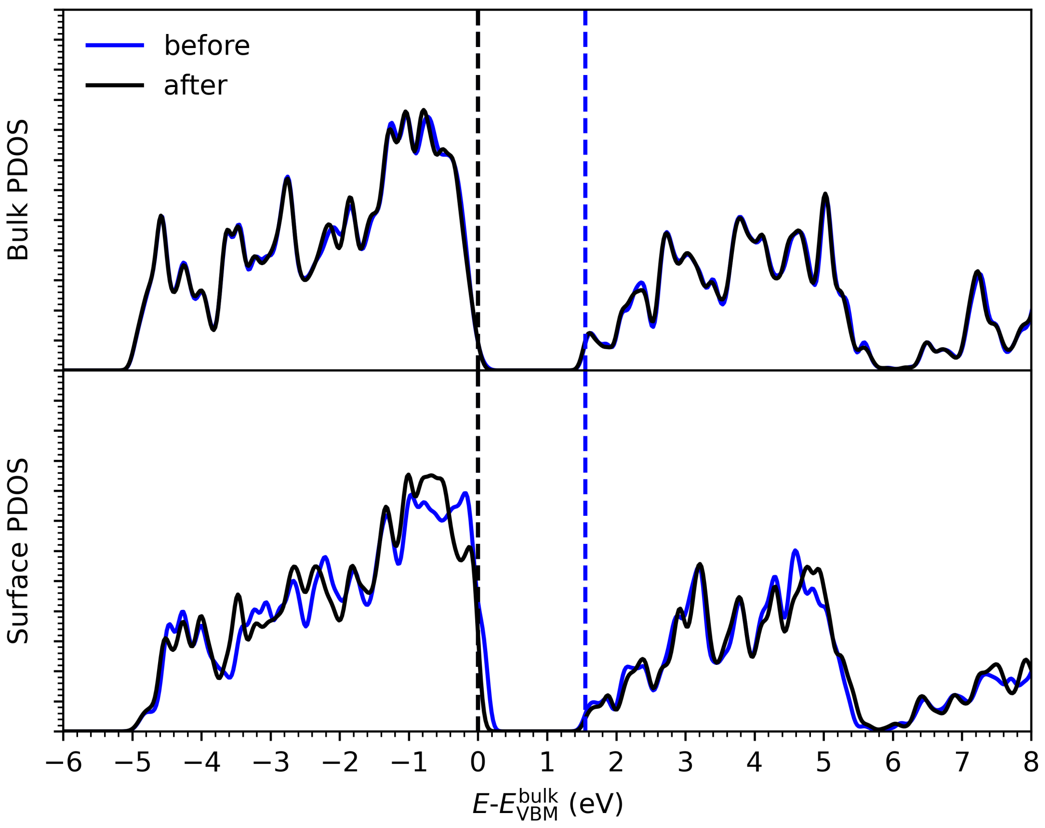

Figure S11: Density of states data for the (001) surface of BiSeI, showing PDOS taken at the surface and centre of the slab, both before and after relaxation.

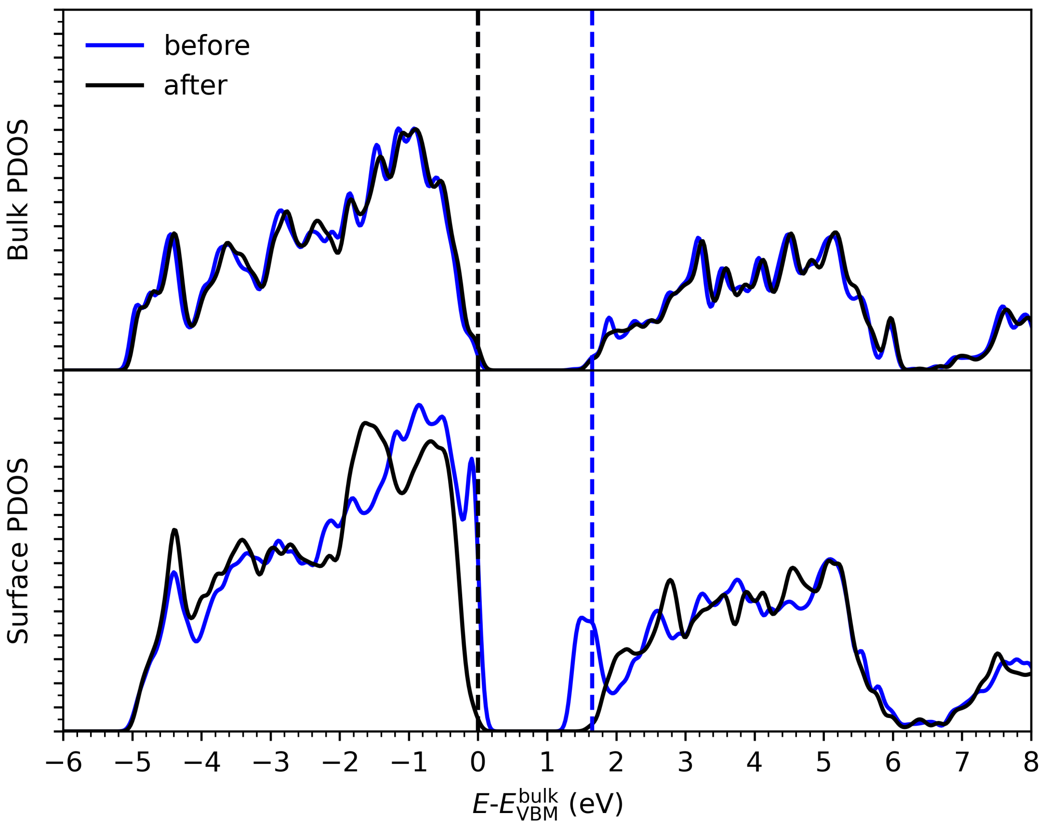

Figure S12: Density of states data for the (100) surface of BiSBr, showing PDOS taken at the surface and centre of the slab, both before and after relaxation.

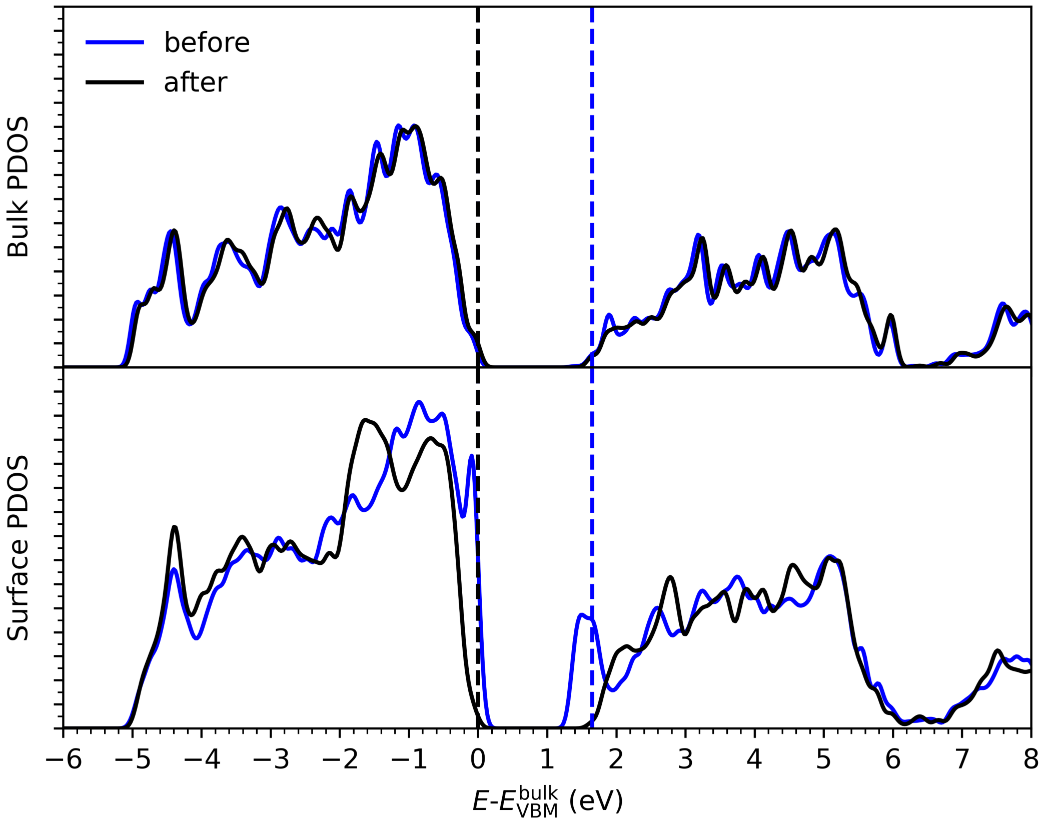

Figure S13: Density of states data for the (100) surface of BiSeBr, showing PDOS taken at the surface and centre of the slab, both before and after relaxation.

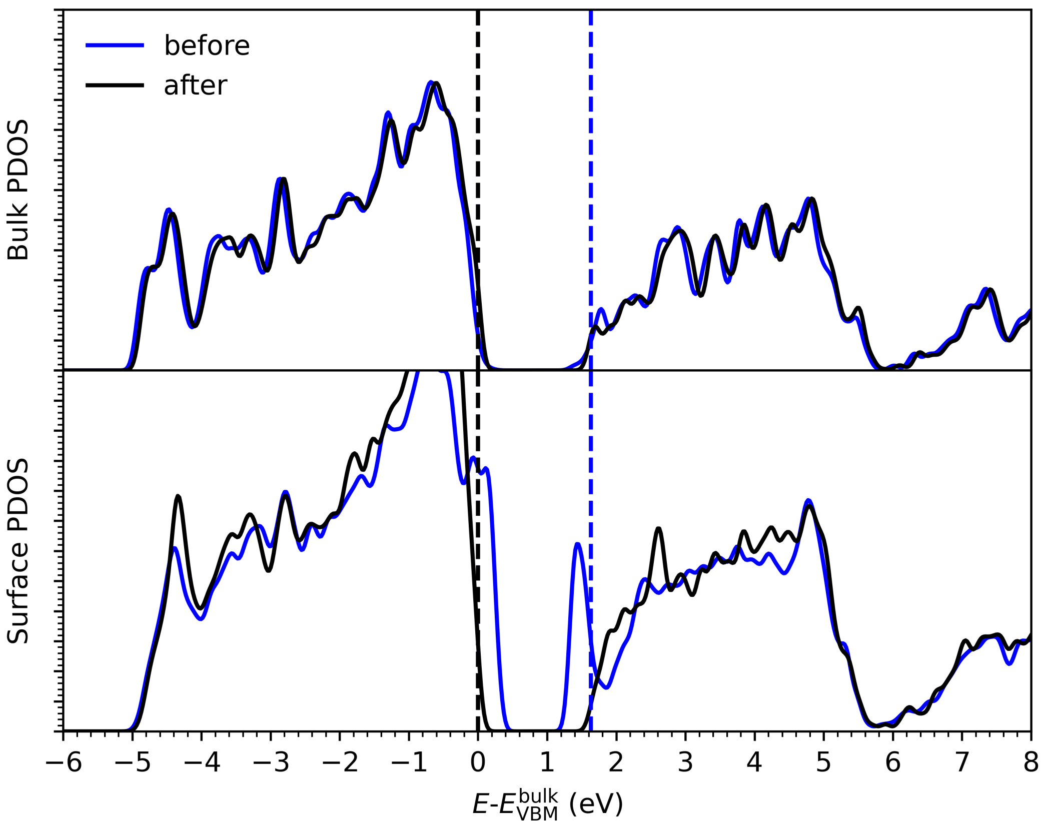

Figure S14: Density of states data for the (100) surface of BiSI, showing PDOS taken at the surface and centre of the slab, both before and after relaxation.

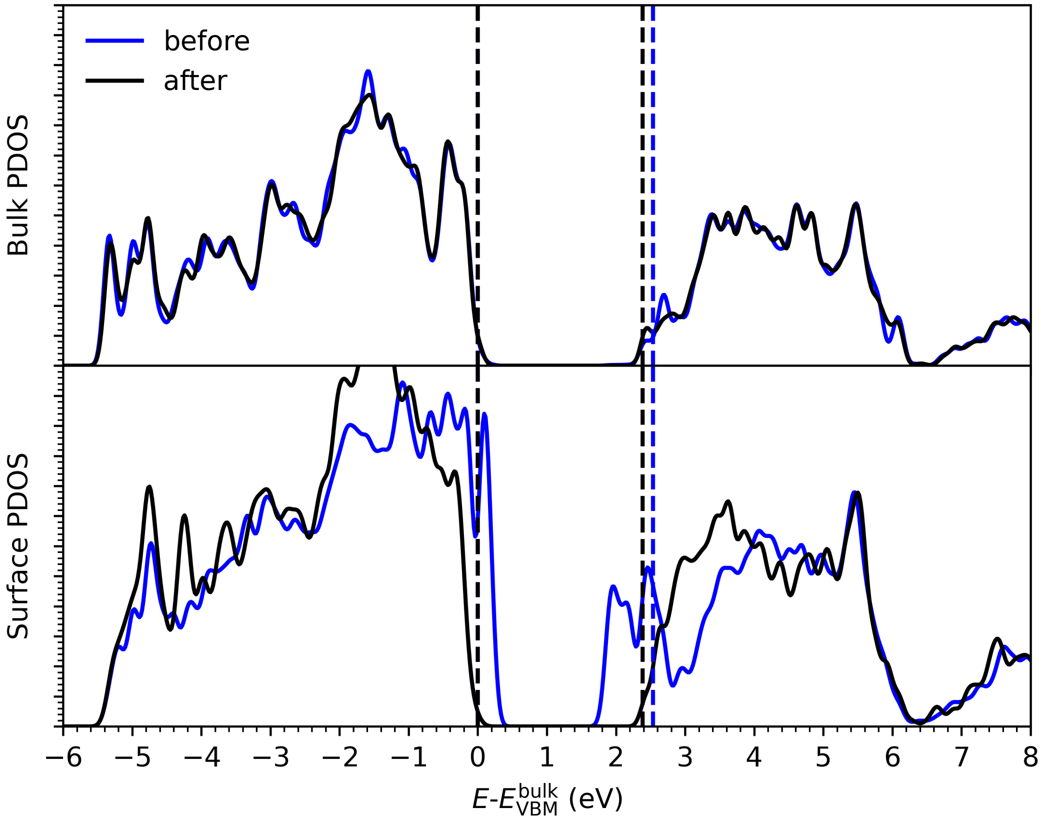

Figure S15: Density of states data for the (100) surface of SbSBr, showing PDOS taken at the surface and centre of the slab, both before and after relaxation.

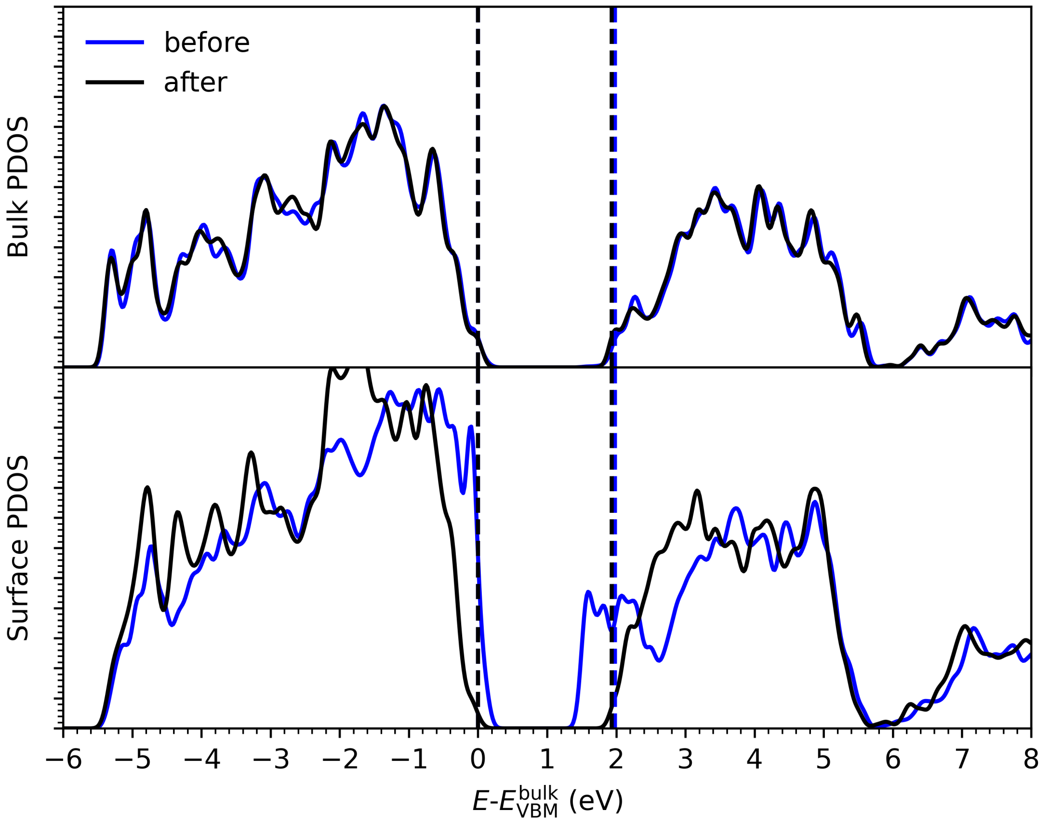

Figure S16: Density of states data for the (100) surface of SbSeBr, showing PDOS taken at the surface and centre of the slab, both before and after relaxation.

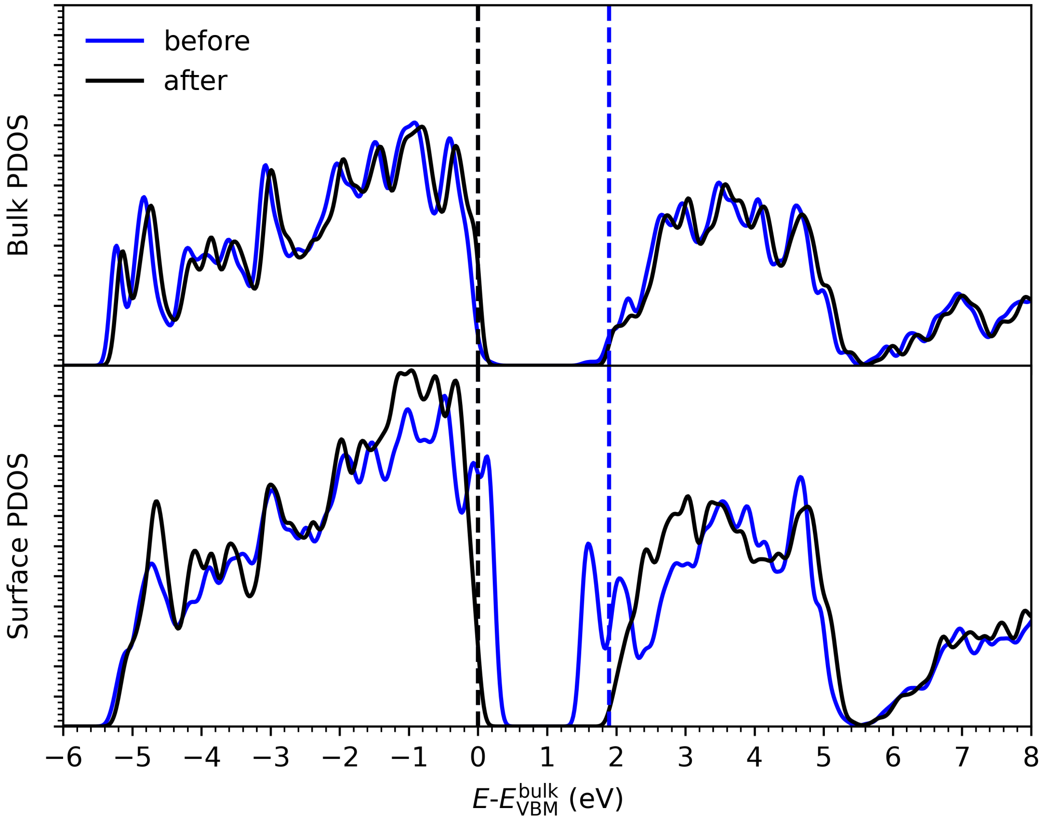

Figure S17: Density of states data for the (100) surface of SbSeI, showing PDOS taken at the surface and centre of the slab, both before and after relaxation.

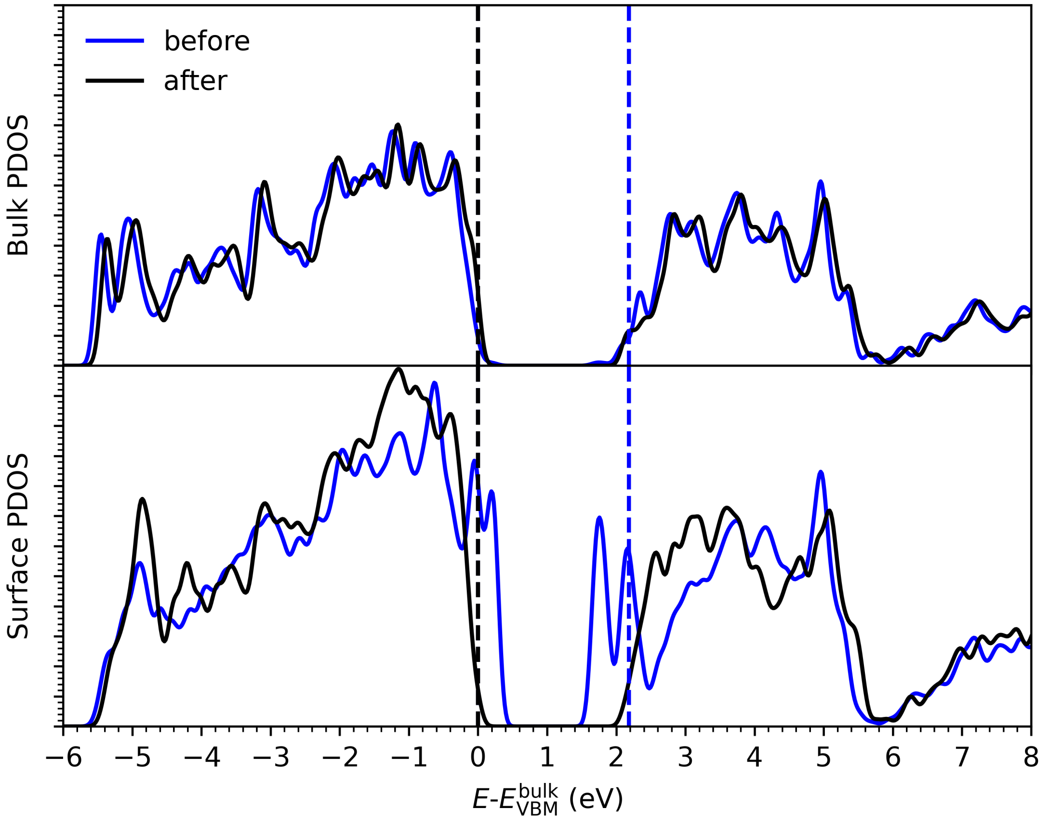

Figure S18: Density of states data for the (100) surface of SbSI, showing PDOS taken at the surface and centre of the slab, both before and after relaxation.

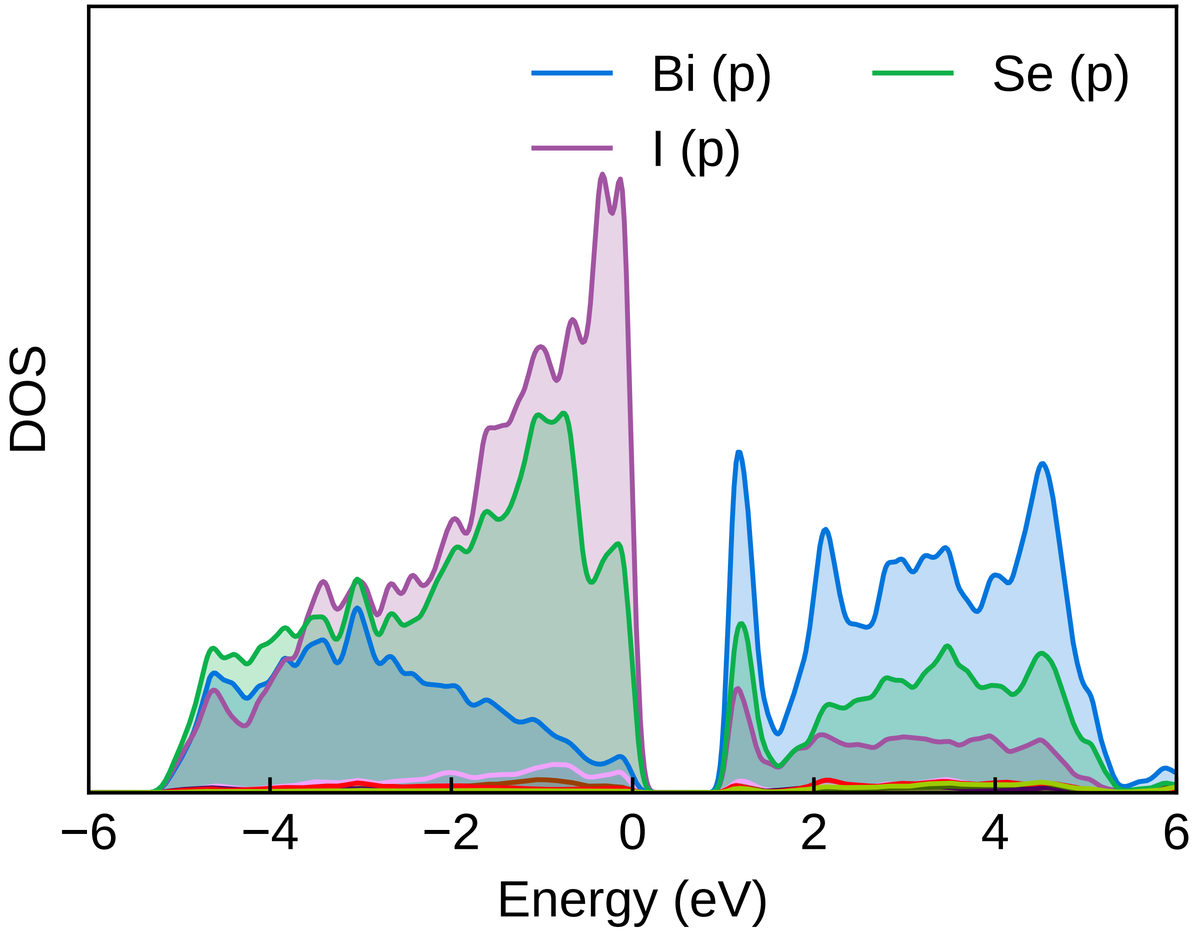

Figure S19: Density of states data for the (100) surface of BiSeI, showing PDOS taken at the surface and center of the slab, broken down via contribution from each atomic species
