# Supplementary material for: Structural and Electronic Reconstruction of Extended Defects in Pnictogen Chalcohalides
Source: J Phys Chem Lett. 2026 Mar 4;17(11):3142–51. doi: 10.1021/acs.jpclett.5c03107 (PMC13007012; doi:10.1021/acs.jpclett.5c03107)
Supplement: Supplementary file 2 [file jz5c03107_si_002.pdf]

jz-2025-031078.R1

Name: Peer Review Information for "Structural and Electronic Reconstruction of Extended Defects in Pnictogen Chalcohalides"

First Round of Reviewer Comments

Reviewer: 1

Comments to the Author

In their study “Structural and Electronic Reconstruction of Extended Defects in Pnictogen Chalcohalides”, the authors Lynch et al. investigate a total of eight different pnictogen chalcohalides using DFT. In my humble opinion, the study has some significant design flaws that may cause confusion to the reader; see my major issues below. Nevertheless, I think the topic is of general interest. I therefore suggest a major revision before a final decision can be made on publication in Journal of Physical Chemistry Letters.

Major issues:

a) One characteristic that appears throughout the study is the comparison between unrelaxed and relaxed structures. The unrelaxed structure is more or less completely irrelevant. Of course, the choice of the unrelaxed structure influences the result of the relaxed structure, but apart from that, an unrelaxed structure is chosen completely arbitrarily and has no scientific relevance whatsoever. In this context, especially when it comes to comparing different surface orientations, it would be much better to compare relaxed bulk structures and relaxed surface structures. Such a comparison would be much more meaningful than the one used so far. In Figure 3, the authors show that the DOS in the bulk hardly changes between unrelaxed and relaxed, but changes significantly at the surface. This is of course the case, since a reconstruction of the surface takes place because the unrelaxed structure is completely arbitrary... However, a comparison in Figure 3 between (relaxed) bulk and surface would be much more interesting, as it would clearly show that something is happening at the VBM and CBM and that the band gap at the

surface is becoming smaller. A pDOS would be well suited for this purpose to describe more precisely which orbital contributions contribute to the VBM and CBM (at the bulk level as well as at the surface level).

Also related to this is the discussion of the inter-chain M-X bond length within the (100) surface, which currently focuses on the unrelaxed structure… In my opinion, it would make more sense to compare the inter-chain M-X bond length at the surface with that at the bulk material… as this way the significance is more or less irrelevant.

b) I don't know if it's just me, but I have a fundamental problem with the term “extended defect”. The authors should not misunderstand me; of course, a surface is a defect of the pure bulk system… but it is the most natural thing in the world… since everything has an end somewhere. When I think of defects, I think of point defects such as vacancies, interstitial lattices, etc., or, in the context of surfaces, of adatom, vacancies, steps, etc. But the authors do not examine any of this; instead, they look at a surface and label it as defective. I think this term is misleading… I would recommend that the authors reconsider using the term ‘extended defects’ for surfaces that are de facto defect-free and revise their text and title accordingly.

c) What I absolutely cannot understand is the use of so many different XC functionals. For one thing, it is not clear to me why the authors perform structure relaxation with PBE+D3 and HSEsol+D3… Especially since the authors then also note the following triviality: “This shows that while PBE+D3 is a far less computationally expensive functional choice, and is known to systemically underestimate band gaps, for structural optimization it is still a reasonable approximation Pnictogen Chalcohalides .” Yes, of course, that's why PBE is still the workhorse in materials science… I understand that the authors want to overcome the known problems of PBE for the corresponding electronic structure, but why they don't simply perform PBE relaxation and HSEsol single points on top? What I also don't understand in this context is that in Figure 1, the authors wrote: “Corresponding band structure calculated using HSEsol+D3+SOC and the PBE+D3 optimized geometry.” That is precisely what I suggested… but if the authors do have HSEsol+D3 structure relaxation data, why don't they calculate a single point with SOC on top for the electronic structure, instead of using the PBEsol+D3 relaxed structure?! Could the authors please explain this in detail?

d) I have been working with VASP and Grimme's DFT-D methods for quite some time, but what I have been wondering is whether DFT-D3 (with regard to S8, A1 and A2 parameters) is parameterised for HSEsol? I know that it is not parameterised for all functionalities available in VASP; I mean, it is parameterised for PBE0, but I am unsure about HSEsol. Could the authors comment on this? If it is not parameterised, what parameters did the authors use? If they did not specify anything within INCAR, the corresponding PBE parameters are stored as default values. The authors should at least mention this in the comp. details.

e) I was wondering why the inter-chain M-X bond only appears on the (100) surface. To me (based on the images), it looks more like a VESTA artifact than a real bond. Would the authors comment on this and verify if necessary? One possibility would be to check the charge transfers.

f) In general, it would certainly be interesting to investigate the charge transfer between bulk and surface within the eight different MchX compounds. Could the authors comment on whether they have considered this and, if so, why they did not pursue this idea further?

g) The authors wrote the following regarding surface preparation: “We found that a slab thickness of at least 30 Å, ...” and “A vacuum gap of 15 Å is employed throughout which minimises interactions between the periodically repeated slabs”. Again, it is difficult for me to assess this based solely on the images, but from Figures S1 and S2, I would guess that the authors' surfaces consist of 6 and 8 layers of M2Ch2X4 (it should be something like that ;)) – The authors may define a layer differently than I do, which is also fine :) - The vacuum, on the other hand, consists of no more than 4 of these units. This seems rather small to me... Could the authors comment on this? Did the authors examine larger vacuums? I am only familiar with this from my own research on coin metal surfaces, where we usually have a ratio of at least 1:2, or rather 1:4, i.e. 1 surface layer to 2/4 vacuum layers (de facto unfilled surfaces). Furthermore, could the authors indicate whether a dipole correction (along the [100] direction) has been used?

Minor issues:

h) In the methods section the authors wrote “gamma centered Monkhorst-Pack” in this case, the authors should use the corresponding Greek gamma.

i) In general, I personally think the transition from bulk MChX structure to surface structures is rather abrupt and difficult to follow. Maybe the authors could take another glance at this and adjust it if necessary.

j) As the authors already have relaxation data for PBEsol+D3 and HSEsol+D3, I would nevertheless focus exclusively on one of the two XCs in the main manuscript and include the values for the other in the SI, since in my opinion they do not contribute anything to the narrative of MchX materials and otherwise only reveal trivialities of DFT…

k) In my opinion, the comparison between unrelaxed and relaxed surfaces in Table 2 is irrelevant, see my point a). A comparison between the energetically preferred surface and the two other surfaces would be more relevant here.

Reviewer: 2

Comments to the Author

The manuscript submitted by Lynch and McKenna presents a computational study focused on defects in pnictogen chalcohalides, a class of materials that remains relatively unexplored despite its emerging and promising potential. The paper is clearly written, engaging, and well structured. The authors employ several computational approaches, ranging from standard GGA (PBE) to hybrid functionals (HSE) including spin-orbit coupling where appropriate. The conclusions appear consistent with the computational findings.

However, I have several comments and suggestions that could help strengthen the manuscript before publication:

1. K-mesh density

a. The k-point meshes used in the calculations are not sufficiently dense to avoid possible electronic discrepancies. I strongly recommend employing at least a k-mesh ensuring a minimum of four k-points in the irreducible Brillouin zone (IBZ).

b. As a result of the current sampling, the density of states (DOS) plots give the impression that the compound behaves as a nearly degenerate semiconductor due to integration artefacts. With four k-points in the IBZ, the authors could use ISMEAR = -4 or -5, which would prevent such effects. I fully acknowledge that this increases the computational cost, but it would also prevent non-specialists from misinterpreting the DOS results.

2. Charge states of defects

Unless I missed it, the authors do not seem to have considered the possible charge states of defects, which is a critical aspect in defect physics (see e.g., works by Van de Walle, Walsh, Zunger, and others). A short discussion and possibly a test case illustrating this effect would significantly enhance the impact of the paper.

3. Computational cost

As a computational chemist, I believe it would be very informative to include a short discussion on the computational cost associated with each level of theory (geometry relaxation, single-point calculations, etc.). Quantifying how much more expensive HSE+SO calculations are compared to standard PBE ones, while yielding potentially similar qualitative trends, would provide valuable insight for the community.

Minor comment:

For this type of study, I often find that the CRYSTAL package, which employs localized Gaussian-type orbitals, offers an efficient alternative to plane-wave approaches by avoiding the high vacuum-related computational cost particularly when using hybrid functionals. If the authors have access to this code, a brief comparison of (i) computational cost and (ii) accuracy versus the VASP results would represent a valuable addition to the work.

In summary, I find the manuscript scientifically sound and clearly presented.

With the above minor revisions and clarifications, I believe it will be suitable for publication in J. Phys. Chem. Lett.

Author's Response to Peer Review Comments:

## <u>Response to Reviewer Comments:</u>

### Reviewer 1:

a) One characteristic that appears throughout the study is the comparison between unrelaxed and relaxed structures. The unrelaxed structure is more or less completely irrelevant. Of course, the choice of the unrelaxed structure influences the result of the relaxed structure, but apart from that, an unrelaxed structure is chosen completely arbitrarily and has no scientific relevance whatsoever. In this context, especially when it comes to comparing different surface orientations, it would be much better to compare relaxed bulk structures and relaxed surface structures. Such a comparison would be much more meaningful than the one used so far. In Figure 3, the authors show that the DOS in the bulk hardly changes between unrelaxed and relaxed, but changes significantly at the surface. This is of course the case, since a reconstruction of the surface takes place because the unrelaxed structure is completely arbitrary… However, a comparison in Figure 3 between (relaxed) bulk and surface would be much more interesting, as it would clearly show that something is happening at the VBM and CBM and that the band gap at the surface is becoming smaller. A pDOS would be well suited for this purpose to describe more precisely which orbital contributions contribute to the VBM and CBM (at the bulk level as well as at the surface level).

Also related to this is the discussion of the inter-chain M-X bond length within the (100) surface, which currently focuses on the unrelaxed structure… In my opinion, it would make more sense to compare the inter-chain M-X bond length at the surface with that at the bulk material… as this way the significance is more or less irrelevant.

Response: Firstly, we would like to clarify the origin of the unrelaxed structures of the surfaces, to clear up any misunderstanding. They are not arbitrability defined: they are constructed by creating a slab from an optimized bulk unit cell. While such an unrelaxed surface is non-physical, in the sense it could not be realized experimentally, it is a welldefined reference configuration for analyzing the effect of surface relaxation on structure, formation energy, and electronic properties. In fact, this type of analysis is extremely common in the literature (1–19).

The reviewer suggested “a comparison in Figure 3 between (relaxed) bulk and surface would be much more interesting”. However, since the unrelaxed surface is constructed from an optimized bulk unit cell the atomic structure and projected density of states of the bulk region of the slab is almost indistinguishable from those of the bulk unit cell. Indeed, we explicitly verified our slab was thick enough to ensure this was the case. However, in the revised manuscript we include a direct comparison to the bulk density of states in the supporting information.

The reviewer also suggested that a partial DOS for both the surface and the bulk could help to describe the VBM and CBM in more detail. This information was already included in the

supporting information (Fig. S19) for the unrelaxed BiSeI surface in order to show the orbital contributions to the gap states.

The reviewer suggested that it would be better to compare the inter-chain M-X bond length at the surface with that in the bulk. However, as discussed above, since the unrelaxed surface is constructed from the optimised bulk structure all comparisons in the paper between relaxed and unrelaxed surfaces structures geometries are exactly equivalent to comparing to the optimized bulk structure. To make this point clearer in the revised manuscript we add the following text to page 5: *"The bulk unit cells optimized using PBE+D3 are used to construct surface (100), (010) and (001) surface slab models for all eight chalcohalide materials. Surface formation energies are calculated both before and after structural optimization"*

b) I don't know if it's just me, but I have a fundamental problem with the term "extended defect". The authors should not misunderstand me; of course, a surface is a defect of the pure bulk system… but it is the most natural thing in the world… since everything has an end somewhere. When I think of defects, I think of point defects such as vacancies, interstitial lattices, etc., or, in the context of surfaces, of adatom, vacancies, steps, etc. But the authors do not examine any of this; instead, they look at a surface and label it as defective. I think this term is misleading… I would recommend that the authors reconsider using the term 'extended defects' for surfaces that are de facto defect-free and revise their text and title accordingly.

Response: The reviewer questions the use of the term "extended defect" to encompass surfaces. Strictly defects should be defined as deviations from the bulk crystal with perfect translational symmetry. Extended defects are those deviations which have extension in one or more dimension. Therefore surface defects are naturally classed as 2D extended defects and the use of the term in this way can be found in many previous papers (20–22). For this reason the use of the term is unlikely to cause any confusion for readers and we prefer to retain it.

c) What I absolutely cannot understand is the use of so many different XC functionals. For one thing, it is not clear to me why the authors perform structure relaxation with PBE+D3 and HSEsol+D3… Especially since the authors then also note the following triviality: "This shows that while PBE+D3 is a far less computationally expensive functional choice, and is known to systemically underestimate band gaps, for structural optimization it is still a reasonable approximation Pnictogen Chalcohalides ." Yes, of course, that's why PBE is still the workhorse in materials science… I understand that the authors want to overcome the known problems of PBE for the corresponding electronic structure, but why they don't simply perform PBE relaxation and HSEsol single points on top? What I also don't understand in this context is that in Figure 1, the authors wrote: "Corresponding band structure calculated using HSEsol+D3+SOC and the PBE+D3 optimized geometry." That is

precisely what I suggested... but if the authors do have HSEsol+D3 structure relaxation data, why don't they calculate a single point with SOC on top for the electronic structure, instead of using the PBEsol+D3 relaxed structure?! Could the authors please explain this in detail?

Response: As described in the manuscript we optimize bulk structures using both PBE+D3 and HSEsol+D3 and quantify the accuracy of predicted lattice constants compared to experiment. We also then perform HSEsol+D3+SOC single point calculations for both bulk structures in order to quantify the accuracy of predicted band gaps. Critically this allows us to also quantify the effect of using a PBE+D3 optimized structure for the HSEsol+D3+SOC calculation of band gap. We conclude that this does lead to any significant error in this case and therefore use the approach for the surface calculations where HSEsol+D3 geometry optimization would be too costly. So as the reviewer notes we already do what they are suggesting. But we have carefully justified that approach by performing comparisons for the bulk structures rather than assuming it would work (in some cases we know it does not so it is important to check (23)).

d) I have been working with VASP and Grimme's DFT-D methods for quite some time, but what I have been wondering is whether DFT-D3 (with regard to S8, A1 and A2 parameters) is parameterised for HSEsol? I know that it is not parameterised for all functionalities available in VASP; I mean, it is parameterised for PBE0, but I am unsure about HSEsol. Could the authors comment on this? If it is not parameterised, what parameters did the authors use? If they did not specify anything within INCAR, the corresponding PBE parameters are stored as default values. The authors should at least mention this in the comp. details.

Response: We specified in the methods section of the paper: “utilizing parameters taken from the Simple DFT-D3 library” (followed by a reference to this library). In the revised manuscript we also include the parameters used in the methods section “*The values used for the van der Waals damping parameters, $a_1$, $a_2$, and $s_8$, are 0.4650, 6.2003, and 2.9215, respectively*” Additionally, it is worth noting that the parameters used provide structural results for the bulk unit cells in very close agreement with experimental data (for the materials where this exists). For example, the lattice parameters for BiSeI (the exemplar materials throughout the paper) obtained in the paper, and experimentally recorded data for these values, are shown in the table below.

| | PBE+D3 | HSEsol+D3 | Experimental value (24) |
|---|---|---|---|
| a | 4.217 | 4.120 | 4.22 |
| b | 8.667 | 8.430 | 8.70 |

| | | | |
|---|---|---|---|
| c | 10.522 | 10.220 | 10.58 |

e) I was wondering why the inter-chain M-X bond only appears on the (100) surface. To me (based on the images), it looks more like a VESTA artifact than a real bond. Would the authors comment on this and verify if necessary? One possibility would be to check the charge transfers.

Response: As stated in the manuscript: “For analysis of the structural changes it is helpful to define a distance below which we consider a strong bond to have formed. For this purpose we take the average of the longest intra-chain bond and the shortest inter-chain bond of each type as a cut-off distance”. This is the length to which the VESTA bond length is set and hence VESTA shows these as bonds, as they fall within the definition we have specified for a new bond. Furthermore, comparing the changes in bond length for the (100) surface to the (001) and (100) surfaces, (100) experiences a change in M-X bond length of approximately ~10% (see Table 2. In the paper). However, for (010) and (001) surfaces, the interatomic spacings change by ~0.5%. We believe that this, in addition to the far smaller surface energy change on relaxation, is evidence that the (001) and (010) surfaces reconstruct without bond formation, whereas the (100) surface behaves noticeably differently due in part to the formation of this new bond.

f) In general, it would certainly be interesting to investigate the charge transfer between bulk and surface within the eight different MchX compounds. Could the authors comment on whether they have considered this and, if so, why they did not pursue this idea further?

Response: There is no drive for charge transfer between bulk and surface regions of the slabs as the systems we are modelling are undoped and stoichiometric. Analyzing the charge before and after relaxation confirms that this is the case – with the surface and the bulk both maintaining bulk-like charges.

g) The authors wrote the following regarding surface preparation: “We found that a slab thickness of at least 30 Å, ...” and “A vacuum gap of 15 Å is employed throughout which minimises interactions between the periodically repeated slabs”. Again, it is difficult for me to assess this based solely on the images, but from Figures S1 and S2, I would guess that the authors' surfaces consist of 6 and 8 layers of $M_2Ch_2X_4$ (it should be something like that ;)) – The authors may define a layer differently than I do, which is also fine :) - The vacuum, on the other hand, consists of no more than 4 of these units. This seems rather small to me... Could the authors comment on this? Did the authors examine larger vacuums? I am only familiar with this from my own research on coin metal surfaces, where we usually have a ratio of at least 1:2, or rather 1:4, i.e. 1 surface layer to 2/4 vacuum layers (de facto

unfilled surfaces). Furthermore, could the authors indicate whether a dipole correction (along the [100] direction) has been used?

Response: All slabs have a thickness of at least 30 Å and the vacuum gap of at least 15 Å. As mentioned in the methods, slab thicknesses of 10 to 50 Å and vacuum gaps of 10 to 25 Å were tested (the latter was not explicitly mentioned in the previous manuscript) in order to confirm that the dimensions we use ensured sufficient accuracy. A dipole correction was not applied to the slab as we model an uncharged stoichiometric and symmetrically terminated slab (maintaining centrosymmetry) and hence no dipole correction is needed.

In the revised manuscript we have added the following to methods section: "*Similarly, vacuum gap thicknesses of 10 Å to 25 Å were tested, and a vacuum gap of 15 Å was found to be sufficient to minimize interactions between the periodically repeated slabs".* We also noticed that the caption to Fig. 2 does not highlight that only the surface layers of the slab are shown. To remedy this, in the revised manuscript we add the following to the caption for Fig 2: *"For the [010] projection, only the outermost six layers (L1-L6) of atoms from one side of the slab are shown".*

h) In the methods section the authors wrote "gamma centered Monkhorst-Pack" in this case, the authors should use the corresponding Greek gamma.

Response: Thank you for spotting this error, which has been corrected in the revised manuscript: on page nine "*charge densities associated with the specific bands in the gap at the Γ point"*, and in the methods section "*Structural optimizations employed a 6 x 3 x 2 Γ centered Monkhorst-Pack k-point grid*".

i) In general, I personally think the transition from bulk MChX structure to surface structures is rather abrupt and difficult to follow. Maybe the authors could take another glance at this and adjust it if necessary.

Response: Thank you for bringing this to our attention. A smoother transition has been written for this section, which also includes an explanation of the surfaces being constructed from an optimized unit cell in order to help prevent the confusion noted in point (a) above.

j) As the authors already have relaxation data for PBEsol+D3 and HSEsol+D3, I would nevertheless focus exclusively on one of the two XCs in the main manuscript and include the values for the other in the SI, since in my opinion they do not contribute anything to the narrative of MchX materials and otherwise only reveal trivialities of DFT...

Response: See response to point (c) above which makes it clear we do not have relaxation data for the surface slabs using HSEsol+D3.

k) In my opinion, the comparison between unrelaxed and relaxed surfaces in Table 2 is irrelevant, see my point a). A comparison between the energetically preferred surface and the two other surfaces would be more relevant here.

Response: See response to point (c) above which explains why this comparison is useful and widely employed in the literature.

**Reviewer 2:**

The manuscript submitted by Lynch and McKenna presents a computational study focused on defects in pnictogen chalcohalides, a class of materials that remains relatively unexplored despite its emerging and promising potential. The paper is clearly written, engaging, and well structured. The authors employ several computational approaches, ranging from standard GGA (PBE) to hybrid functionals (HSE) including spin-orbit coupling where appropriate. The conclusions appear consistent with the computational findings.

However, I have several comments and suggestions that could help strengthen the manuscript before publication:

1. K-mesh density

a. The k-point meshes used in the calculations are not sufficiently dense to avoid possible electronic discrepancies. I strongly recommend employing at least a k-mesh ensuring a minimum of four k-points in the irreducible Brillouin zone (IBZ).

Response: The smallest k-point mesh used in the paper for the surface calculations contains eight k-points in the irreducible Brillouin zone. As such, the K-point density is higher than the reviewer's suggested minimum mesh-density.

b. As a result of the current sampling, the density of states (DOS) plots give the impression that the compound behaves as a nearly degenerate semiconductor due to integration artefacts. With four k-points in the IBZ, the authors could use ISMEAR = -4 or -5, which would prevent such effects. I fully acknowledge that this increases the computational cost, but it would also prevent non-specialists from misinterpreting the DOS results.

Response: The density of states within the paper does not show that the compound acts as nearly degenerate semiconductor. The reviewer seems to have misinterpreted dashed lines on the DOS plot, which are intended to show the band edges. This confusion, however, is in part due to ambiguity in the paper, which does not clearly explain the purpose of the dashed lines. We thank the reviewer for bringing this to our attention, and to make this point clearer in the revised manuscript we have updated the DOS plot in Fig. 3

and added the following text to the caption: *"The dashed lines indicate the positions of the band edges for the bulk region."*

2. Charge states of defects

Unless I missed it, the authors do not seem to have considered the possible charge states of defects, which is a critical aspect in defect physics (see e.g., works by Van de Walle, Walsh, Zunger, and others). A short discussion and possibly a test case illustrating this effect would significantly enhance the impact of the paper.

Response: This is a stoichiometric surface, and as such the only possible charge state is neutral in order to maintain overall neutrality. Changing the charge would correspond to ionization or electron capture by the surface, which would not be within the scope of the current study.

3. Computational cost

As a computational chemist, I believe it would be very informative to include a short discussion on the computational cost associated with each level of theory (geometry relaxation, single-point calculations, etc.). Quantifying how much more expensive HSE+SO calculations are compared to standard PBE ones, while yielding potentially similar qualitative trends, would provide valuable insight for the community.

Response: We thank the reviewer for this feedback. In the revised manuscript, a small discussion has been added to the methods section, which highlights the relative computational costs for the various types of calculations performed in the study.

Minor comment:

For this type of study, I often find that the CRYSTAL package, which employs localized Gaussian-type orbitals, offers an efficient alternative to plane-wave approaches by avoiding the high vacuum-related computational cost particularly when using hybrid functionals. If the authors have access to this code, a brief comparison of (i) computational cost and (ii) accuracy versus the VASP results would represent a valuable addition to the work.

Response: Whilst this would perhaps be interesting, a comparison between different DFT packages exceeds the scope of this work. Similarly, one could argue this about any DFT software of the readers preference. We actually prefer CP2K as an alternative to CRYSTAL if we would like to use Gaussian-type orbitals.

1. Frankcombe TJ, Løvvik OM. The Crystal Structure and Surface Energy of NaAlH4: A Comparison of DFT Methodologies. J Phys Chem B. 2006 Jan 1;110(1):622–30.
2. Fajín JLC, Illas F, Gomes JRB. Effect of the exchange-correlation potential and of surface relaxation on the description of the H2O dissociation on Cu(111). J Chem Phys. 2009 Jun 10;130(22):224702.
3. Santos-Carballal D, Roldan A, Grau-Crespo R, Leeuw NH de. A DFT study of the structures, stabilities and redox behaviour of the major surfaces of magnetite Fe3O4. Phys Chem Chem Phys. 2014 Sep 17;16(39):21082–97.
4. Viñes F, Sousa C, Liu P, Rodriguez JA, Illas F. A systematic density functional theory study of the electronic structure of bulk and (001) surface of transition-metals carbides. J Chem Phys. 2005 May 5;122(17):174709.
5. Filippetti A, Fiorentini V, Cappellini G, Bosin A. Anomalous relaxations and chemical trends at III-V semiconductor nitride nonpolar surfaces. Phys Rev B. 1999 Mar 15;59(12):8026–31.
6. Wolf D. Correlation between energy, surface tension and structure of free surfaces in fcc metals. Surface Science. 1990 Feb 2;226(3):389–406.
7. Castleton CWM, Höglund A, Mirbt S. Density functional theory calculations of defect energies using supercells. Modelling Simul Mater Sci Eng. 2009 Nov;17(8):084003.
8. Spencer MJS, Hung A, Snook IK, Yarovsky I. Density functional theory study of the relaxation and energy of iron surfaces. Surface Science. 2002 Jul 1;513(2):389–98.
9. Mele EJ, Joannopoulos JD. Electronic states at unrelaxed and relaxed GaAs (110) surfaces. Phys Rev B. 1978 Feb 15;17(4):1816–27.
10. Ivanov I, Pollmann J. Electronic structure of ideal and relaxed surfaces of ZnO: A prototype ionic wurtzite semiconductor and its surface properties. Phys Rev B. 1981 Dec 15;24(12):7275–96.
11. Mukhopadhyay AB, Sanz JF, Musgrave CB. First-principles calculations of structural and electronic properties of monoclinic hafnia surfaces. Phys Rev B. 2006 Mar 24;73(11):115330.
12. Liu W, Zheng WT, Jiang Q. First-principles study of the surface energy and work function of III-V semiconductor compounds. Phys Rev B. 2007 Jun 22;75(23):235322.
13. Sun YY, Xu H, Feng YP, Huan ACH, Wee ATS. Multilayer relaxations of (3 1 1), (3 3 1) and (2 1 0) fcc transition metal surfaces studied by pseudopotential DFT calculations. Surface Science. 2004 Jan 1;548(1):309–16.
14. Dzade NY, Leeuw NH de. Periodic DFT+U investigation of the bulk and surface properties of marcasite (FeS2). Phys Chem Chem Phys. 2017 Oct 18;19(40):27478–88.
15. Guan L, Li X, Li Q, Guo J, Jin L, Zhao Q, et al. Relaxation and electronic states of Au(100), (110) and (111) surfaces. Solid State Communications. 2009 Oct 1;149(37):1561–4.

16. Singh-Miller NE, Marzari N. Surface energies, work functions, and surface relaxations of low-index metallic surfaces from first principles. Phys Rev B. 2009 Dec 7;80(23):235407.

17. Kwon SK, Nabi Z, Kádas K, Vitos L, Kollár J, Johansson B, et al. Surface energy and stress release by layer relaxation. Phys Rev B. 2005 Dec 22;72(23):235423.

18. Keller M, Belabbes A, Furthmüller J, Bechstedt F, Botti S. Surface properties of hexagonal Si and Ge: First-principles study of energetics, atomic relaxation, and electronic structure. Phys Rev B. 2025 Nov 7;112(19):195303.

19. Nichols RJ, Nouar T, Lucas CA, Haiss W, Hofer WA. Surface relaxation and surface stress of Au(1 1 1). Surface Science. 2002 Jul 1;513(2):263–71.

20. Mrovec M, Gröger R, Bailey AG, Nguyen-Manh D, Elsässer C, Vitek V. Bond-order potential for simulations of extended defects in tungsten. Phys Rev B. 2007 Mar 30;75(10):104119.

21. de Leeuw NH, Higgins FM, Parker SC. Modeling the Surface Structure and Stability of αQuartz. J Phys Chem B. 1999 Feb 1;103(8):1270–7.

22. Extended Defects in Semiconductors and Their Interactions with Point Defects and Impurities. In: Physical Chemistry of Semiconductor Materials and Processes [Internet].
John Wiley & Sons, Ltd; 2015 [cited 2025 Dec 9]. p. 195–264. Available from: https://onlinelibrary.wiley.com/doi/abs/10.1002/9781118514610.ch3

23. Tong CJ, Edwards HJ, Hobson TDC, Durose K, Dhanak VR, Major JD, et al. Density Functional Theory and Experimental Determination of Band Gaps and Lattice Parameters in Kesterite Cu2ZnSn(SxSe1–x)4. J Phys Chem Lett. 2020 Dec 17;11(24):10463–8.

24. Trifonov VA, Shevel'kov AV, Dikarev EV, Popovkin BA. Crystal Structure of SbSeBr and BiSeI from X-ray Powder Diffraction. Russ J Inorg Chem. 1999 Jan;44(1):5–8.

jz-2025-031078.R2

Name: Peer Review Information for "Structural and Electronic Reconstruction of Extended Defects in Pnictogen Chalcohalides"

Second Round of Reviewer Comments

Reviewer: 1

Comments to the Author

I still have fundamental concerns regarding the manuscript and find the authors' line of argumentation difficult to follow. While reading Ref. 14 (Advanced Electronic Materials 2021, 7, 2000908) helped to some extent, a manuscript should be self-contained and comprehensible without requiring detailed familiarity with the authors' prior work. My main concern is the insufficient communication of the study's motivation and of key computational details. Although the methodology appears robust, several design choices are not adequately explained, making it difficult to assess the rationale behind the approach. In addition, I am not convinced that the authors are genuinely engaging with the reviewers' critiques. Both reviewers raised similar issues (e.g., regarding the treatment of defects), yet the authors' responses suggest a reluctance to substantially refine or clarify their work. In its current form, I cannot support publication in The Journal of Physical Chemistry Letters. I recommend a further major revision, contingent on the authors' willingness to significantly sharpen the presentation of their motivation, methodological choices, and overall narrative, so that the core message of the study becomes clear and accessible to the reader.

Major issues:

a) I would like to return to the term "extended defects", which both reviewers have found problematic. I find it remarkable that the authors refer to a pristine surface as an "extended defect", and I continue to consider this terminology misleading. Toward the end of the manuscript, the authors state: "First, while current predictions are based on vacuum conditions, surface reconstructions are likely to differ under realistic environments, such as in the presence of water, oxygen, or during photo-electrochemical operation." Based on this statement, as well as on Ref. 14, I infer that the authors may primarily be interested in interfaces and internal boundaries within the active layer of a solar cell. However, since modelling the full interface may be computationally to demanding, therefore the surface exposed to vacuum is treated as a proxy and is therefore referred to as an "extended defect". The authors may correct me if this interpretation is inaccurate, but this is how I understand their responses to both reviewers and the cited passages. In the same context, the authors further wrote: "We also note that the present study addresses ideal chalcohalide surfaces exposed to a vacuum in order to understand the intrinsic properties of such extended defects. When surfaces are exposed to an environment, such as in

photo-electrochemical cells, surface composition and structure can be affected by interaction with various chemical species (e.g., from solutions or the atmosphere) which would require extension on the models presented here." If my interpretation above is correct, this would also help clarify the motivation for the present study. In any case, the authors must provide a clear and explicit definition of what they mean by "extended defects", ideally at the very beginning of the manuscript. If surfaces are being used as a model for interfaces or grain boundaries, this needs to be stated unambiguously. Moreover, the authors should explain why such "extended defects" are relevant for MChX systems, particularly in light of their concluding statement: "This positions pnictogen chalcohalides as promising candidates for polycrystalline thin-film photovoltaics." A precise definition and a clearer discussion of the physical relevance of the term "extended defects" are essential for understanding the scope, motivation, and implications of the study.

b) First, I would like to firmly reject the authors' implicit suggestion that my previous comment under point a), regarding the use of unrelaxed structures, reflects a lack of understanding on my part. I believe my remark concerning "arbitrariness" was intentionally misconstrued. Naturally, a surface constructed from a bulk structure, whether relaxed or unrelaxed, is not arbitrary per se. Nevertheless, an unrelaxed surface has only limited physical relevance, as such a configuration would not occur in nature. Independent of this apparent miscommunication, the motivation for comparing unrelaxed and relaxed surfaces remains unclear to me. The authors state that this is a well-established approach, but primarily cite references that are approximately 20 years old (with the notable exception of Silvana's work). In the revised manuscript, the authors merely state: "Surface formation energies are calculated both before and after structural optimization (Table 2)." However, this still does not explain the reason for the comparison between relaxed and unrelaxed surface structures… Referring to Ref. 14, the authors wrote: "The difference between the unrelaxed SFE (i.e., the formation energy of the as-cleaved surface) and the fully optimized surface (Δ) is an useful measure of the degree of relaxation." The authors should please correct me if I am wrong, but I understand that the authors' motivation for the unrelaxed/relaxed comparison is to show how the bulk structure changes at an interface/grain boundary. Personally, I still think that a direct comparison between bulk and surface (both relaxed!) is more suitable for this purpose, but I am no expert in this field and am open to be proven wrong. Could the authors comment on whether my assumption in this regard is correct?

c) I am still not convinced that the comparison between PBE+D3 and HSEsol+D3 is meaningful for the bulk structure, as the authors also arrive at the following trivial conclusion: "This shows that while PBE+D3 is a far less computationally expensive functional choice, and is known to systemically underestimate band gaps, for structural optimization it is still a reasonable approximation." as well as "This confirms that using PBE+D3 geometries and HSEsol+D3+SOC for optoelectronic properties is a reasonable approach to employ for surfaces (where the cost of full optimization using HSEsol+D3 would be too great)." As the authors are surely aware, it is standard practice to optimise with PBE/PBEsol and subsequently determine the electronic structure using single-point hybrid calculations. It is still unclear to me why the authors think that the comparison between PBE and HSEsol is of such essential importance that it must be included in the main text. I could understand it being in the SI and one sentence in the main text, but Table 1 and the subsequent discussion revolve around this triviality… Could the authors explain their motivation in more detail at this point?

d) In addition, in response to my question about the DFT-D3 parameters, the authors added the following: "The values used for the van der Waals damping parameters, a1, a2, and s8 , are 0.4650, 6.2003, and 2.9215, respectively." Firstly, I would like to thank the authors for pointing out that the DFT-D3 is parametrised for HSEsol. On the other hand, the authors are surely aware that these are the parameters for HSEsol. I hope the authors have not also used these for PBE. Could the authors clarify this? In my previous question, I overlooked the fact that the authors referred to the DFT-D3 library, which answered my question already. However, in the revised manuscript, this is definitely misleading – which was not my intention!

e) I must admit that I still find the description of the computational details for the surface relaxations difficult to follow. In particular, I am confused by the choice of k-point grids at different stages of the calculations. For the PBE surface relaxations, the authors state: "For the structural optimizations of these surfaces, a Monkhorst–Pack k-point grid of 6 x 3 x 1 was chosen for (001) surfaces, 6 x 2 x 1 for the (010) surfaces, and 3 x 2 x 1 for the (100) surfaces." However, for the subsequent HSEsol single-point calculations, the authors wrote: "A Monkhorst–Pack k-point grid of 4 x 2 x 1 was employed for each of the surfaces, and a plane-wave energy cutoff of 500 eV." My first question concerns the reduction of the k-point density for the HSEsol single-point calculations. Typically, one would expect the k-point sampling to be increased rather than reduced when moving to a higher-level electronic-structure method in order to achieve a more accurate description. Could the

authors please clarify the rationale behind this choice? Second, I do not understand why a single k-point grid is now used for the (100), (010), and (001) surfaces in the HSEsol calculations, whereas different k-point grids were employed for the surface relaxations. Please explain how this change is justified and whether convergence with respect to k-point grid has been verified consistently for all surfaces. Relatedly, the authors state: “For the density of states calculations the Hartree–Fock contributions were calculated using a reduced grid of 2 x 1 x 1 and spin–orbit coupling was included.” It is unclear to me why an even smaller k-point grid was used at this stage, and how this choice affects the reliability of the resulting density of states. Further clarification would be appreciated. Finally, the paragraph as a whole is difficult to follow, particularly the statement: “The density of states of the surfaces before and after optimization were computed using HSEsol+D3+SOC with the PBE+D3 optimized structures.” My understanding is: PBE+D3 (optimisation) -> PBE+D3+SOC (single point) -> HSEsol+D3+SOC (single point) + something with HF?! The authors should clarify which structures were used for which calculations and in what sequence. In addition, please specify the alpha value used in the HSEsol calculations – I would assume the default 25% HF exchange?

f) With regard to my point b) and Table 2: If my assumption is correct that the authors regard the comparison between unrelaxed and relaxed as an indicator of relaxation, as suggested by the following statement: “However, during relaxation the surface energy decreases dramatically, falling by an average of 36%.” Wouldn't a delta value between unrelaxed and relaxed in Table 2 be useful? Could the authors comment on this?

g) With regard to the density of states (DOS) and the comparison between unrelaxed and relaxed structures, the authors state: “A density of states projection for the (001) and (010) surfaces reveals that these surfaces do not generate any gap states, either before, or after, reconstruction (see Fig. S10 and S11 for BiSeI).” It is unclear to me why the comparison between unrelaxed and relaxed surfaces is relevant in this context, given that the unrelaxed surface has no direct physical relevance, as it would not occur in nature. As mentioned in my previous comment (point b), the unrelaxed structure should be regarded as a computational “auxiliary construct”, whose purpose is, at best, to facilitate relaxation toward the true ground-state geometry. While I can understand such a comparison in the context of bulk structures, extending this analysis to surfaces, and, moreover, presenting the DOS for both unrelaxed and relaxed bulk structures, appears even less meaningful from a physical standpoint. I therefore struggle to see the scientific value of these comparisons. Could the authors please clarify their motivation in more detail and explain why they

believe that comparing the DOS of unrelaxed and relaxed bulk and surface structures provides relevant physical insight?

h) With regard to my point g), I first note an apparent inconsistency in the authors' statements. On the one hand, the authors wrote: "The projected density of states in the surface region (L1-2) shows that before relaxation a gap state is formed via the cleavage of bonds at the surface. However, after relaxation we can see that this gap state disappears; i.e., the reconstruction has eliminated the gap state" On the other hand, they state: "A density of states projection for the (001) and (010) surfaces reveals that these surfaces do not generate any gap states, either before, or after, reconstruction" These two statements appear contradictory to me. More fundamentally, even if a gap state were indeed present in the unrelaxed surface, I do not understand its physical or computational relevance. The unrelaxed surface does not represent a realistic/experimentel structure and, according to the authors' own description, the gap state vanishes upon relaxation. It therefore remains unclear why the presence of a gap state in an unrelaxed, nonphysical configuration should be considered meaningful or worthy of discussion. Could the authors please clarify both the apparent inconsistency in their statements and, more importantly, explain the motivation for highlighting electronic states that exist only in the unrelaxed surface but disappear in the physically relevant, relaxed structure?

Minor issues:

i) In connection with point c), could the authors explain why they use HSEsol but not PBEsol? I don't understand the mixture of a hybrid optimised for solids with an GGA functional which is "unoptimised for solids". (At least it is not specifically optimised for solids, such as PBEsol, for example.)

j) In their response, the authors referred to me, as well as to Figure S19 in the manuscript, which is not included in the SI. I assume they mean S18 here.

Author's Response to Peer Review Comments:

Reviewer(s)' Comments to Author:

Reviewer: 1

Recommendation: This paper may be publishable, but major revision is needed; I would like to be invited to review any future revision.

Comments:
I still have fundamental concerns regarding the manuscript and find the authors' line of argumentation difficult to follow. While reading Ref. 14 (Advanced Electronic Materials 2021, 7, 2000908) helped to some extent, a manuscript should be self-contained and comprehensible without requiring detailed familiarity with the authors' prior work. My main concern is the insufficient communication of the study's motivation and of key computational details. Although the methodology appears robust, several design choices are not adequately explained, making it difficult to assess the rationale behind the approach. In addition, I am not convinced that the authors are genuinely engaging with the reviewers' critiques. Both reviewers raised similar issues (e.g., regarding the treatment of defects), yet the authors' responses suggest a reluctance to substantially refine or clarify their work. In its current form, I cannot support publication in The Journal of Physical Chemistry Letters. I recommend a further major revision, contingent on the authors' willingness to significantly sharpen the presentation of their motivation, methodological choices, and overall narrative, so that the core message of the study becomes clear and accessible to the reader.

We are very happy to answer queries on scientific issues and address any issues with the presentation. We always do so honestly and professionally and any misunderstanding or misinterpretation that may have occurred in our previous response is absolutely not deliberate or any indication of a lack of proper engagement.

Major issues:
a) I would like to return to the term "extended defects", which both reviewers have found problematic. I find it remarkable that the authors refer to a pristine surface as an "extended defect", and I continue to consider this terminology misleading. Toward the end of the manuscript, the authors state: "First, while current predictions are based on vacuum conditions, surface reconstructions are likely to differ under realistic environments, such as in the presence of water, oxygen, or during photo-electrochemical operation." Based on this statement, as well as on Ref. 14, I infer that the authors may primarily be interested in interfaces and internal boundaries within the active layer of a solar cell. However, since modelling the full interface may be computationally to demanding, therefore the surface exposed to vacuum is treated as a proxy and is therefore referred to as an "extended defect". The authors may correct me if this interpretation is inaccurate, but this is how I

understand their responses to both reviewers and the cited passages. In the same context, the authors further wrote: "We also note that the present study addresses ideal chalcohalide surfaces exposed to a vacuum in order to understand the intrinsic properties of such extended defects. When surfaces are exposed to an environment, such as in photoelectrochemical cells, surface composition and structure can be affected by interaction with various chemical species (e.g., from solutions or the atmosphere) which would require extension on the models presented here." If my interpretation above is correct, this would also help clarify the motivation for the present study. In any case, the authors must provide a clear and explicit definition of what they mean by "extended defects", ideally at the very beginning of the manuscript. If surfaces are being used as a model for interfaces or grain boundaries, this needs to be stated unambiguously. Moreover, the authors should explain why such "extended defects" are relevant for MChX systems, particularly in light of their concluding statement: "This positions pnictogen chalcohalides as promising candidates for polycrystalline thin-film photovoltaics." A precise definition and a clearer discussion of the physical relevance of the term "extended defects" are essential for understanding the scope, motivation, and implications of the study.

We checked again and could find no comments from reviewer 2 indicating an issue with the use of the term "extended defects" and they note that "the paper is clearly written, engaging, and well structured". The reviewer is correct in their interpretation that part of our motivation is to use surface defects as a proxy for internal interfaces like grain boundaries. The properties of surface defects are also interesting in their own right. To address the concerns above in the revised manuscript we have inserted additional text on page 3 to explain what we mean extended defect and unambiguously explain the motivations of the study as follows:

"Since pnictogen chalcohalide thin films are typically polycrystalline understanding the properties of grain boundary defects is important since they are often performance limiting for PV applications (as in the case of CdTe discussed above). In this letter we use the term "extended defect" in the broadest possible sense to include any disruption of the order of an infinite periodic crystal with extension in one or more dimensions.[26] Viewed in this way surfaces and grain boundaries are both examples of two-dimensional extended defects. In fact surfaces and grain boundaries share many similar features beyond their dimensionality such as modification of atom coordination and bonding, a strain field which decays with distance from the surface/grain boundary plane and a modified electronic structure compared to the bulk crystal. Indeed in many semiconducting materials the electronic properties of surfaces and grain boundaries are found to be closely related.[27] Therefore, while modeling the properties of surfaces is of interest in their own right it can also provide an indication of the likely properties of grain boundary defects but with much reduced computational complexity, as demonstrated in our previous work on $Sb_2Se_3$.[14-16] In

this study we model the structure and properties of surfaces in the pnictogen chalcohalide materials to provide insight into their electronic properties and by extension that of grain boundary defects."

b) First, I would like to firmly reject the authors' implicit suggestion that my previous comment under point a), regarding the use of unrelaxed structures, reflects a lack of understanding on my part. I believe my remark concerning "arbitrariness" was intentionally misconstrued. Naturally, a surface constructed from a bulk structure, whether relaxed or unrelaxed, is not arbitrary per se. Nevertheless, an unrelaxed surface has only limited physical relevance, as such a configuration would not occur in nature. Independent of this apparent miscommunication, the motivation for comparing unrelaxed and relaxed surfaces remains unclear to me. The authors state that this is a well-established approach, but primarily cite references that are approximately 20 years old (with the notable exception of Silvana's work). In the revised manuscript, the authors merely state: "Surface formation energies are calculated both before and after structural optimization (Table 2)." However, this still does not explain the reason for the comparison between relaxed and unrelaxed surface structures... Referring to Ref. 14, the authors wrote: "The difference between the unrelaxed SFE (i.e., the formation energy of the as-cleaved surface) and the fully optimized surface (Δ) is an useful measure of the degree of relaxation." The authors should please correct me if I am wrong, but I understand that the authors' motivation for the unrelaxed/relaxed comparison is to show how the bulk structure changes at an interface/grain boundary. Personally, I still think that a direct comparison between bulk and surface (both relaxed!) is more suitable for this purpose, but I am no expert in this field and am open to be proven wrong. Could the authors comment on whether my assumption in this regard is correct?

We reiterate that any misunderstanding or misinterpretation that may have occurred in our previous response was absolutely not deliberate. We engaged genuinely with the reviews and tried our best to interpret and respond to them.

In the revised manuscript we add a more detailed motivation and explanation on page 6.

"The atoms in the "before" configuration are positioned as they would be in a perfect bulk crystal. In the "after" configuration the atoms near the surface displace to adopt a lower energy structure. The difference in atom positions between the "before" and "after" configurations represents the strain field induced by the surface which diminishes with distance from the surface (the slab models should be sufficiently thick that the displacements in the center of the slab are very small, which we have verified is the case). The formation energy of the surface after structural optimization is the one relevant for

predictions of surface stability but the change in the formation energy on structural optimization provides additional insight, with larger differences indicative of a more significant reconstruction, transforming a low stability termination into a more stable one.[48-50]"

c) I am still not convinced that the comparison between PBE+D3 and HSEsol+D3 is meaningful for the bulk structure, as the authors also arrive at the following trivial conclusion: "This shows that while PBE+D3 is a far less computationally expensive functional choice, and is known to systemically underestimate band gaps, for structural optimization it is still a reasonable approximation." as well as "This confirms that using PBE+D3 geometries and HSEsol+D3+SOC for optoelectronic properties is a reasonable approach to employ for surfaces (where the cost of full optimization using HSEsol+D3 would be too great)." As the authors are surely aware, it is standard practice to optimise with PBE/PBEsol and subsequently determine the electronic structure using single-point hybrid calculations. It is still unclear to me why the authors think that the comparison between PBE and HSEsol is of such essential importance that it must be included in the main text. I could understand it being in the SI and one sentence in the main text, but Table 1 and the subsequent discussion revolve around this triviality... Could the authors explain their motivation in more detail at this point?

We address this point in the revised manuscript by adding the following on page 4:

"Accurate prediction of the electronic properties of semiconductors typically requires either many-body perturbation theory or hybrid functionals (both with inclusion of spin-orbit coupling). The former is prohibitively expensive for large supercells such as those employed here. The latter is costly, but feasible if one can first obtain the structure using a less expensive exchange-correlation approximation. Unfortunately, there are known cases where employing this shortcut leads to inaccurate predictions.[28,29] Therefore, it is essential to carefully validate the approach by comparing self-consistently optimized hybrid functional calculations of structure and band gap with the less expensive two-step approach.

d) In addition, in response to my question about the DFT-D3 parameters, the authors added the following: "The values used for the van der Waals damping parameters, a1, a2, and s8 , are 0.4650, 6.2003, and 2.9215, respectively." Firstly, I would like to thank the

authors for pointing out that the DFT-D3 is parametrised for HSEsol. On the other hand, the authors are surely aware that these are the parameters for HSEsol. I hope the authors have not also used these for PBE. Could the authors clarify this? In my previous question, I overlooked the fact that the authors referred to the DFT-D3 library, which answered my question already. However, in the revised manuscript, this is definitely misleading – which was not my intention!

We employ the standard PBE DFT-D3. In the revised manuscript we make it explicit that the parameters quoted in the manuscript are for HSEsol:

"The values used for the van der Waals damping parameters for HSEsol are $a_1 = 0.4650$, $a_2 = 6.2003$ and $s_8 = 2.9215$."

e) I must admit that I still find the description of the computational details for the surface relaxations difficult to follow. In particular, I am confused by the choice of k-point grids at different stages of the calculations. For the PBE surface relaxations, the authors state: "For the structural optimizations of these surfaces, a Monkhorst–Pack k-point grid of 6 x 3 x 1 was chosen for (001) surfaces, 6 x 2 x 1 for the (010) surfaces, and 3 x 2 x 1 for the (100) surfaces." However, for the subsequent HSEsol single-point calculations, the authors wrote: "A Monkhorst–Pack k-point grid of 4 x 2 x 1 was employed for each of the surfaces, and a plane-wave energy cutoff of 500 eV." My first question concerns the reduction of the kpoint density for the HSEsol single-point calculations. Typically, one would expect the kpoint sampling to be increased rather than reduced when moving to a higher-level electronic-structure method in order to achieve a more accurate description. Could the authors please clarify the rationale behind this choice? Second, I do not understand why a single k-point grid is now used for the (100), (010), and (001) surfaces in the HSEsol calculations, whereas different k-point grids were employed for the surface relaxations. Please explain how this change is justified and whether convergence with respect to k-point grid has been verified consistently for all surfaces. Relatedly, the authors state: "For the density of states calculations the Hartree–Fock contributions were calculated using a reduced grid of 2 x 1 x 1 and spin–orbit coupling was included." It is unclear to me why an even smaller k-point grid was used at this stage, and how this choice affects the reliability of the resulting density of states. Further clarification would be appreciated. Finally, the paragraph as a whole is difficult to follow, particularly the statement: "The density of states of the surfaces before and after optimization were computed using HSEsol+D3+SOC with the PBE+D3 optimized structures." My understanding is: PBE+D3 (optimisation) -> PBE+D3+SOC (single point) -> HSEsol+D3+SOC (single point) + something with HF?! The authors should clarify which structures were used for which calculations and in what

sequence. In addition, please specify the alpha value used in the HSEsol calculations – I would assume the default 25% HF exchange?

A single k-point grid was not used for all of the surfaces. The ambiguity arose as the methods mainly focuses on the (100) surfaces as the main results presented in the manuscript. But of course, we did compute properties for the (010) and (100) too and so should specify the parameters for those calculations too.

The very high computational cost of the HSEsol+D3+SOC calculations means we are unable to increase the k-point grids significantly compared to PBE+D3 but we are able to keep them comparable. For the HSEsol calculations as is common practice we evaluate the exact exchange contributions using a less dense grid (NKRED option in VASP) but all other contributions are evaluated using the more dense grid.

In the revised manuscript we have made some edits to make it clearer which k-point grids are used for which surfaces and the sequence of calculations. We also explicitly state the Hartree-Fock percentage used in HSEsol:

"The density of states of the surfaces before and after optimization were computed using HSEsol+D3+SOC (with 25% Hartree-Fock exchange) using the PBE+D3 optimized structures.
For the HSEsol+D3+SOC calculations the k-point grids used were 6 x 2 x 1 for (001) surfaces, 6 x 2 x 1 for the (010) surfaces, and 4 x 2 x 1 for the (100) surfaces. These slightly modified grid sizes were chosen to be divisible by two to allow for down-sampling of the exact exchange contributions on smaller grids of 3 x 1 x 1 for (001) surfaces, 3 x 1 x 1 for the (010) surfaces, and 2 x 1 x 1 for the (100) surfaces (using the NKRED option in VASP)."

and

"So the full sequence of calculations is optimization using PBE+D3, a single point calculation using PBE+D3+SOC and a single point using HSEsol+D3+SOC (where the exact exchange contributions are evaluated on a coarser k-point grid)."

f) With regard to my point b) and Table 2: If my assumption is correct that the authors regard the comparison between unrelaxed and relaxed as an indicator of relaxation, as suggested by the following statement: "However, during relaxation the surface energy decreases dramatically, falling by an average of 36%." Wouldn't a delta value between unrelaxed and relaxed in Table 2 be useful? Could the authors comment on this?

Yes, that is the correct assumption. In the revised manuscript we have added the Delta value for the (100) surface to Table 2. We could add for the other surfaces as well but the table would become quite wide.

g) With regard to the density of states (DOS) and the comparison between unrelaxed and relaxed structures, the authors state: "A density of states projection for the (001) and (010) surfaces reveals that these surfaces do not generate any gap states, either before, or after, reconstruction (see Fig. S10 and S11 for BiSeI)." It is unclear to me why the comparison between unrelaxed and relaxed surfaces is relevant in this context, given that the unrelaxed surface has no direct physical relevance, as it would not occur in nature. As mentioned in my previous comment (point b), the unrelaxed structure should be regarded as a computational "auxiliary construct", whose purpose is, at best, to facilitate relaxation toward the true ground-state geometry. While I can understand such a comparison in the context of bulk structures, extending this analysis to surfaces, and, moreover, presenting the DOS for both unrelaxed and relaxed bulk structures, appears even less meaningful from a physical standpoint. I therefore struggle to see the scientific value of these comparisons. Could the authors please clarify their motivation in more detail and explain why they believe that comparing the DOS of unrelaxed and relaxed bulk and surface structures provides relevant physical insight?

In the revised manuscript we add a more detailed motivation and explanation on page 8.

"To analyse the electronic structure of the surfaces we compute the density states (DOS) for the slabs in the unrelaxed bulk-like ("before") and relaxed ("after") configurations and project the densities of states in both near surface and bulk regions (i.e., the center of the slab). Analogous to the analysis of structure and stability presented above, comparing the electronic structure for these different configurations provides additional insight beyond computation of the relaxed structure alone. For example, for a sufficiently thick slab (such that the strain in the central bulk region is small) the DOS projected in the bulk region should be very similar before and after relaxation and equivalent to that of a 3D periodic bulk crystal. For all the calculations we have performed we have verified this is the case. A DOS projection in the surface region for the (001) and (010) surfaces reveals that these surfaces do not generate any gap states, either before, or after, relaxation (see Fig. S10 and S11 for BiSeI). However, the behavior of the (100) surface is very different as shown in Figure 3a for BiSeI. The projected density of states in the surface region (L1-2) shows that before relaxation a gap state is formed via the cleavage of bonds at the surface. However, after relaxation we can see that this gap state disappears; i.e., the reconstruction has eliminated (or self-healed) the gap state. This same effect is observed in all of the materials

investigated (see Fig. S12-18). It shows that the specific reconstruction of this surface is essential for eliminating the gap state, which is not a conclusion one could make on the basis of analyzing the "after" configuration only."

h) With regard to my point g), I first note an apparent inconsistency in the authors' statements. On the one hand, the authors wrote: "The projected density of states in the surface region (L1-2) shows that before relaxation a gap state is formed via the cleavage of bonds at the surface. However, after relaxation we can see that this gap state disappears; i.e., the reconstruction has eliminated the gap state" On the other hand, they state: "A density of states projection for the (001) and (010) surfaces reveals that these surfaces do not generate any gap states, either before, or after, reconstruction" These two statements appear contradictory to me. More fundamentally, even if a gap state were indeed present in the unrelaxed surface, I do not understand its physical or computational relevance. The unrelaxed surface does not represent a realistic/experimentel structure and, according to the authors' own description, the gap state vanishes upon relaxation. It therefore remains unclear why the presence of a gap state in an unrelaxed, nonphysical configuration should be considered meaningful or worthy of discussion. Could the authors please clarify both the apparent inconsistency in their statements and, more importantly, explain the motivation for highlighting electronic states that exist only in the unrelaxed surface but disappear in the physically relevant, relaxed structure?

The gap state is present for the (100) surface but not for the other two surfaces. We believe the additional explanation and revisions made in response to point g above have made this clearer.

Minor issues:

i) In connection with point c), could the authors explain why they use HSEsol but not PBEsol? I don't understand the mixture of a hybrid optimised for solids with an GGA functional which is "unoptimised for solids". (At least it is not specifically optimised for solids, such as PBEsol, for example.)

We tested various exchange correlation approximations for these materials and found PBE to give a better prediction of bulk lattice parameters than PBEsol and so adopted this approach.

j) In their response, the authors referred to me, as well as to Figure S19 in the manuscript, which is not included in the SI. I assume they mean S18 here.

Unfortunately an older version of the supporting information file was uploaded in the last revision by mistake. The correct Figure is S19 but it was missing. In the revised submission we include the correct supporting information file.

jz-2025-031078.R3

Name: Peer Review Information for "Structural and Electronic Reconstruction of Extended Defects in Pnictogen Chalcohalides"

Third Round of Reviewer Comments

Reviewer: 1

Comments to the Author

I would like to thank the authors for clarifying previous misconceptions and revising their study. I think the authors have addressed my concerns in detail, e.g. with regard to “extended defects” and the comparison between unrelaxed and relaxed structures, and have explained their motivation for doing so. In my view, there are no longer any objections against publication in The Journal of Physical Chemistry Letters, and I therefore recommend acceptance of the study.

Author's Response to Peer Review Comments:

Dear Editor,

Assessing the recommended non-scientic changes listed, we found that the incomplete references referred to are complete with all the required informtaion. Additionally, TOC

graphic is already included in the paper, titled "TOC-graphic", and labelled as "Figure 1: For Table of Contents Only". As such, no changes have been made to the paper.

With sincere regards,

Thomas Lynch
